# Untargeted Metabolomics Profiling of a PFAS-Exposed Flemish Population

**DOI:** 10.3390/metabo16020135

**Published:** 2026-02-15

**Authors:** María del Mar Delgado-Povedano, Haesong Sher, Leen Jacobs, Maria van de Lavoir, Rani Robeyns, Ann Colles, Eva Govarts, Elly Den Hond, Giulia Poma, Alexander L. N. van Nuijs, Adrian Covaci

**Affiliations:** 1 Toxicological Centre, University of Antwerp, 2610 Antwerp, Belgium; 2 VITO Health, Flemish Institute for Technological Research (VITO), Boeretang 200, 2400 Mol, Belgium; 3 Provincial Institute of Hygiene, Provincial Research Centre for Environment and Health, 2023 Antwerp, Belgium

**Keywords:** polyfluoroalkyl substances, residential exposure, metabolic perturbations, serum profiling

## Abstract

**Background/Objectives**: Per- and polyfluoroalkyl substances (PFAS) are persistent environmental pollutants that accumulate in humans through everyday exposure pathways, raising concern about long-term metabolic health effects in exposed populations. This study aimed to characterize PFAS-associated serum metabolic alterations in a Flemish population residing within a 3 km radius of a PFAS production facility using untargeted metabolomics and lipidomics. **Methods**: A cohort of 82 adults was stratified into high-exposure (*n* = 41, median total PFAS = 162.0 ng/mL) and low-exposure (*n* = 41, median total PFAS = 7.2 ng/mL) groups. Serum metabolic profiling was performed using four liquid chromatography–high-resolution mass spectrometry (LC-HRMS)-based platforms. Univariate and multivariate statistics were conducted to identify metabolites that were differentially expressed between both exposure groups. **Results**: The analysis revealed 38 altered metabolites. Overall, high PFAS exposure was characterized by upregulation of phosphatidylglycerols (PG), phosphatidylinositols, phosphatidylethanolamines (PE), and triacylglycerols (TG) and downregulation of sphingomyelins, with differential regulation of ceramides, hexosylceramides (HexCer), and phosphatidylcholines. Glycerophospholipid metabolism as well as sphingolipid metabolism pathways were identified as perturbed. Seven lipids and one amino acid showed weak-to-strong correlations (|r|= 0.23–0.61) with PFAS levels. A panel of five metabolites was selected to explore whether they collectively form a potential metabolic signature associated with PFAS exposure. This panel, including L-aspartic acid, PG 18:0_18:2, HexCer (d18:1/14:0), PE 16:0_18:3, and TG 16:0_20:5_22:6, showed moderate discrimination between residents with high and low PFAS levels (area under the curve, AUC = 0.753). **Conclusions**: This study identifies coordinated lipid metabolic changes associated with PFAS exposure and highlights a small, exploratory metabolite panel that may provide complementary insight into the biological effects of PFAS.

## 1. Introduction

Per- and polyfluoroalkyl substances (PFAS) are a broad class of synthetic fluorinated chemicals. Specifically, PFAS are defined as fluorinated substances that contain at least one fully fluorinated methyl or methylene carbon atom (without any H/Cl/Br/I atom attached to it), i.e., with a few noted exceptions, any chemical with at least a perfluorinated methyl group (−CF3) or a perfluorinated methylene group (−CF2−) is a PFAS [1]. Since their production began in 1947, they have been widely applied in textiles, food packaging, cookware, electronics, firefighting foams, pesticides, cosmetics, and many other applications [2,3]. Humans are continuously exposed to PFAS through everyday environmental and consumer sources. Their persistence in the environment, their bioaccumulation in some organs, and their mobility have raised concerns for human and environmental health [4].

Bioaccumulation of PFAS in human body tissues has been linked to some adverse health conditions [5]. Toxicological studies in mice have associated high PFAS concentrations with endocrine disruption, delay in physical development, cancer and neonatal mortality [5,6]. A study in zebrafish exposed to PFAS found abnormal ventroflexion of the tail and failed swim bladder inflation [7]. In humans, PFAS have been reported to activate receptors associated with carcinogenesis, e.g., peroxisome proliferator-activated receptors (PPAR), due to their structural resemblance to fatty acids and can disrupt lipid metabolism, resulting in dyslipidemia [5,8]. The binding of PFAS to PPAR has been linked to poor fetal growth and immune function [5].

Untargeted metabolomics is an exploratory analytical strategy designed to profile a wide range of endogenous low-molecular-weight compounds (generally <1500 Da) in biological samples, thereby capturing the biochemical status of a system at a specific time point [9,10,11]. As metabolite levels are closely associated with phenotypic variation, metabolomic profiling offers valuable insight into both physiological and pathological processes. Furthermore, this approach supports the identification of candidate biomarkers, the discovery of pharmacological targets, and the elucidation of molecular mechanisms underlying biological responses [12]. The metabolomic markers identified through these approaches may act as key intermediates for elucidating the complex relationship between environmental exposure, molecular effects, and clinical outcomes [13,14]. Some metabolomics studies in in vivo or in vitro models have associated PFAS exposure with the metabolism of lipids, amino acids and purines [15,16,17], but the dose used in experimental studies might not accurately reflect the exposure levels experienced by the general population. Human studies investigating biological responses associated with PFAS exposure have primarily focused on targeted lipid or hormone markers or biomarkers of liver function [18,19,20]. Also, increasing numbers of epidemiological studies [21,22,23] have used untargeted metabolomics to identify early effect biomarkers for health risks associated with PFAS exposure.

Industrial and manufacturing processes have been the major sources of PFAS contamination in the environment [24]. Belgium has experienced significant industrial PFAS contamination, particularly around Antwerp [24]. Biomonitoring programs have shown that certain populations living in that area exhibited higher serum PFAS concentrations compared to national reference values measured in 2017–2018, indicating potential long-term environmental exposure [25].

Serum and plasma are widely used matrices for PFAS biomonitoring [26] and metabolomics studies, as they reflect biochemical changes and can be readily integrated with epidemiological and clinical metadata.

In this context, the present study aimed to characterize serum metabolic alterations associated with PFAS exposure in a Flemish population (*n* = 82) residing near a PFAS production facility using untargeted metabolomics and lipidomics. The study provides a comprehensive characterization of PFAS-associated metabolic variation and identifies key metabolic features that may offer insight into the biological responses to PFAS exposure.

## 2. Materials and Methods

### 2.1. Materials and Chemicals

Hippuric acid-(phenyl-^13^C_6_), leucine-D_3_, D-glucose-^13^C_6_, cholic acid-D_4_, dodecanoic acid-D_3_, succinic acid-D_4_, sodium pyruvate-^13^C_3_, glutamic acid-D_5_, tryptophan-D_5_, ammonium formate (≥99%, LC-MS grade), ammonium acetate (LC-MS grade) and ammonium carbonate (HPLC grade) were purchased from Sigma-Aldrich (St. Louis, MO, USA). Caffeine-^13^C_3_ was acquired from Cerilliant Corporation (Round Rock, TX, USA). Octanoyl-L-carnitine-(N-methyl-D_3_), ceramide [d18:1/18:1-(9Z)-^13^C_18_] and L-lysine-^13^C_6_-^15^N_2_ were obtained from Cambridge Isotope Laboratories (Andover, MA, USA). The SPLASH™ LIPIDOMIX™ Mass Spec Standard—containing 15:0–18:1 (D_7_) PC, 15:0–18:1 (D_7_) PE, 15:0–18:1 (D_7_) PS, 15:0–18:1 (D_7_) PG, 15:0–18:1 (D_7_) PI, 15:0–18:1 (D_7_) PA, 18:1 (D_7_) LPC, 18:1 (D_7_) LPE, 18:1-D_7_-cholesterol, 18:1 (D_7_) MG, 15:0–18:1 (D_7_) DG, 15:0–18:1 (D_7_)-15:0 TG, 18:1 (D_9_) SM, and cholesterol (D_7_)—as well as acetic acid (100%, LC-MS grade), ammonia solution (25%, LC-MS grade), isopropanol (IPA, ACS grade) and chloroform (CHCl_3_, analytical grade) were purchased from Merck (Darmstadt, Germany). Solvents including methanol (MeOH), acetonitrile (MeCN) and formic acid (HCOOH, 99%), all ULC/MS–CC/SFC grade, were purchased from Biosolve (Valkenswaard, The Netherlands). Ultrapure water (H_2_O, 18.2 MΩ·cm) was obtained using an Elga Pure Lab system (Tienen, Belgium). Nylon centrifugal filters (0.2 μm) were acquired from VWR (Radnor, PA, USA). Pure, dry nitrogen (AZOTE N_28_, N_2_) was obtained from Air Liquide Belge (Liège, Belgium).

### 2.2. Study Cohort

All residents aged ≥12 years living within a 3 km radius of the 3M facility in Zwijndrecht, Belgium (*n* = 12,089), were invited to participate in the “PFAS-bloedonderzoek 2021” human biomonitoring initiative. Recruitment was voluntary via a secure central website of the Flemish government. To ensure geographical representativeness, only one individual per residential address was eligible for participation, and the study area was divided into four predefined zones (one zone within 1.5 km of the PFAS production facility, and three zones between 1.5 and 3 km). To ensure age representativeness, enrolment targets within each zone were stratified into three age groups (12–20, 21–49, and 50–99 years), proportional to the number of residents in each age group. Breastfeeding women were included regardless of age or residential zone. Applicants exceeding the target per zone or age group were placed on a waiting list, and individuals were selected from this list as needed to achieve the recruitment goals.

Of the 947 individuals who applied, 838 were selected to participate, and 804 attended the study visits. Complete data were obtained from 796 participants, including demographic information, biological sex, age, breastfeeding status, financial situation based on income and household education level, which were collected via self-reported online questionnaires. At the time of blood collection, a nurse measured their body weight and height using calibrated equipment, from which body mass index (BMI) was calculated, and recorded the exact blood collection time.

From the 796 total participants, all adults aged 20–59 years (*n* = 517) were used to define the distribution of total PFAS serum concentrations. This age range was chosen to minimize metabolic variation due to age and puberty. Based on this distribution, a subset of 82 participants representing the extremes of PFAS exposure was selected for metabolomics analysis. Specifically, the subset included 41 adults (low-exposure group) from the lowest total PFAS serum concentrations (≤10th percentile) and 41 adults (high-exposure group) from the highest total PFAS serum concentrations (≥90th percentile), making clear that the 82 participants are the sum of both extreme groups. Total PFAS serum concentration was calculated as the sum of the individual PFAS compound concentrations measured in serum samples, including perfluorobutanoic acid (PFBA), perfluoropentanoic acid (PFPeA), perfluorohexanoic acid (PFHxA), perfluoroheptanoic acid (PFHpA), perfluorooctanoic acid (PFOA), perfluorononanoic acid (PFNA), perfluorodecanoic acid (PFDA), perfluoroundecanoic acid (PFUnA), perfluorododecanoic acid (PFDoA), perfluorobutanesulfonate (PFBS), perfluorohexanesulfonate (PFHxS), perfluoroheptanesulfonate (PFHpS) and perfluorooctanesulfonate (PFOS). This extreme-phenotype approach was chosen to maximize contrast between low- and high-exposure levels [27], enhancing the detection of PFAS-associated metabolomic alterations. However, this approach may reduce generalizability to individuals with intermediate exposures and could overestimate effect sizes if extreme groups differ in other characteristics.

### 2.3. Sample Collection

Blood (10 mL) samples were collected once from each participant throughout the day, reflecting daily metabolic variation, into BD Vacutainer^®^ serum tubes (BD; Becton, Dickinson and Company, Franklin Lakes, NJ, USA) between 15th July and 24th August 2021. Collection tubes from a single manufacturer were consistently used for all samples in the study [28,29]. Collected blood samples were transported under refrigerated conditions to VITO (Mol, Belgium). Immediately upon arrival, samples were centrifuged at 1300× *g* for 10 min at room temperature to isolate the serum fraction. This duration is sufficient to allow proper clotting while minimizing residual enzymatic activity and the risk of hemolysis, which could release intracellular metabolites and affect metabolomic profiles. All samples were processed using the same standardized protocol, minimizing potential pre-analytical variability across the cohort [28]. One aliquot of 500 µL was stored in a 1.5 mL cryotube at −80 °C for PFAS analysis, while the remaining serum was stored in a 4 mL cryotube at −80 °C in the biobank [28,30]. For metabolomics analysis, serum samples were retrieved from this biobank storage, thawed once to generate aliquots into Eppendorf^®^ tubes (Eppendorf SE, Hamburg, Germany), stored at −80 °C and shipped to the analyzing lab on dry ice, where they remained frozen until further processing.

Written informed consent was received from all participants, as stated in the file submitted to the Ethical Committee (protocol code 0436—Edge n/a—BUN B3002021000126, approved on 3 August 2023). Participants were informed about the study protocol and signed the informed consent form.

### 2.4. Sample Preparation

A 240 µL aliquot of serum was thawed a second time for quenching by adding 840 µL of ice-cold MeOH (−80 °C) [serum-to-MeOH ratio 1:3.5 (*v*/*v*)] and subsequently stored at −80 °C until extraction. Prior to extraction, samples were thawed on ice and immediately processed. All samples were subjected to two freeze–thaw cycles to reduce inter-sample variability [28]. All serum samples were visually inspected for hemolysis to minimize pre-analytical variation [28]. Eight blanks, prepared from a pool of ultrapure water obtained from five serum tubes, were processed identically to the samples [31]. To reduce technical bias, sample collection, preparation, and injection were all randomized independently [32].

Metabolites were extracted using a modified Folch liquid–liquid extraction (LLE) protocol [33]. Serum samples were mixed with MeOH/H_2_O/CHCl_3_ (4:3:8, *v*/*v*/*v*) at a sample-to-solvent ratio of 1:20 (*v*/*v*) [33]. Specifically, quenched serum samples were transferred to a glass vial for LLE, containing 404 μL of ice-cold MeOH (at −20 °C) and 2560 μL of pre-chilled CHCl_3_ (at −20 °C). Internal standards (ISs) were divided into three mixtures, the first contained 40 μg/mL of hippuric acid-(phenyl-^13^C_6_), L-lysine-^13^C_6_-^15^N_2_, leucine-D_3_, D-glucose-^13^C_6_, glutamic acid-D5, tryptophan-D5, succinic acid-D4, pyruvic acid-^13^C_3_ and caffeine-^13^C_3_ in H_2_O/MeOH (1:1, *v*/*v*). The second contained 50 μg/mL of cholic acid-D4, 20 μg/mL of octanoyl-L-carnitine-(N-methyl-D3), 40 μg/mL of ceramide [d18:1/18:1(9Z)-^13^C_18_] and 10 μg/mL of dodecanoic acid-D3 in CHCl_3_. The third contained 24 μL of SPLASH^TM^ mixture in MeOH. IS mixtures were added to the polar and apolar mixture to obtain a final concentration between 0.2 and 4 μg/mL (depending on the internal standard) after reconstitution. Subsequently, the extraction mixture was vortexed for 60 s, incubated on ice for 30 min, added with 730 μL of ultrapure water, incubated on ice for 10 min, and centrifuged at 9000× *g* for 2 min at room temperature, followed by equilibration at 4 °C for 10 min. The resulting polar and apolar fractions were divided into two subfractions prior to evaporation, to allow analysis of each fraction in both positive and negative polarity during LC-HRMS acquisition.

For the polar fraction (upper phase), a volume of 1460 μL was transferred to an Eppendorf^®^ tube, without compromising the protein layer. After vortexing for 20 s, 730 μL was transferred to a second Eppendorf^®^ tube after which they were evaporated using dry nitrogen at room temperature. A volume of 2800 μL of the apolar fraction (lower phase) was transferred to a 4 mL glass vial using a glass syringe. After vortexing for 20 s, 1400 μL was transferred to a second glass vial and evaporated using dry nitrogen at room temperature. Dried extracts were stored at −80 °C and reconstituted immediately before analysis. Polar extracts were reconstituted in 240 µL of MeCN/H_2_O (65:35, *v*/*v*), and apolar fractions were reconstituted in 100 µL of IPA/MeOH (35:65, *v*/*v*). After vortexing for 40 s, samples were filtered through 0.2 μm nylon centrifugal filters by centrifugation at 14,000× *g* for 2 min at room temperature. All samples remained cooled during acquisition in the autosampler (4 °C). Polar fractions were analyzed using two HILIC methods on the metabolomics platform, whereas apolar fractions were analyzed by RPLC on the lipidomics platform to cover a broad range of lipid classes. Supplementary Materials Figure S1 summarizes in an illustration the serum preparation procedure.

Additionally, equal volumes (15 μL) from each non-blank sample extract were combined into a separate LC-vial to generate a pooled quality control (QC) sample for each analytical platform and ionization mode. A separate pool QC sample was prepared each day using only the samples extracted on that day (≤25) to avoid excessive dilution associated with pooling large numbers of samples. The pooled QC samples were used for analytical system conditioning, MS/MS data acquisition, and to assess analytical precision through seven repeated injections at a predetermined frequency throughout each run [34].

In addition to metabolite extraction, separate serum aliquots (500 µL) were processed for PFAS analysis. The samples were stored at −20 °C for up to 6 weeks for analysis. After thawing and homogenization, isotope-labeled ISs (PFBA-^13^C_4_, PFPeA-^13^C_4_,PFHxA-^13^C_2_, PFOA-^13^C_4_, PFNA-^13^C_5_, PFDA-^13^C_2_, PFUnA-^13^C_2_, PFDoA-^13^C_2_, PFHxS-^18^O, and PFOS-^13^ C_4_) were added. Methanol was used for protein precipitation, and the precipitate was removed. Ten microliters of the resulting supernatant was injected for quantification by UPLC-MS/MS (Waters Corporation, Milford, MA, USA).

### 2.5. PFAS Analysis

PFAS concentrations were quantified using UPLC-MS/MS (Waters Acquity Xevo TQ-(X)S) with an electrospray ionization (ESI) source (Waters Corporation, Milford, MA, USA). The system was equipped with a Waters Acquity UPLC BEH Shield RP18 column (1.7 μm, 2.1 × 100 mm column) and a Waters Vanguard pre-column (Acquity UPLC BEH C18; 2.1 × 5 mm, 1.7 μm). Gradient elution using water- and MeOH-based solvents was performed at 300 μL/min and 40 °C. Measurements were carried out in Multiple Reaction Monitoring (MRM) mode in negative ESI.

The following PFAS were quantified in serum: perfluorobutanoic acid (PFBA), perfluoropentanoic acid (PFPeA), perfluorohexanoic acid (PFHxA), perfluoroheptanoic acid (PFHpA), perfluorooctanoic acid (PFOA), perfluorononanoic acid (PFNA), perfluorodecanoic acid (PFDA), perfluoroundecanoic acid (PFUnA), perfluorododecanoic acid (PFDoA), perfluorobutanesulfonate (PFBS), perfluorohexanesulfonate (PFHxS), perfluoroheptanesulfonate (PFHpS) and perfluorooctanesulfonate (PFOS).

The method was previously validated for a measurement range of 0.2–30 ng/mL PFAS in serum, which was extended to 480 ng/mL for this study. Samples exceeding this range were re-analyzed following appropriate dilution. Each batch included quality control measurements, comprising calibration standards, integration standards for the evaluation of branched isomers, and a control serum sample. The method’s performance was monitored using control charts. The expanded measurement uncertainty of the method (U, k = 2, 95% confidence) ranged from 23 to 50% depending on the PFAS.

Participants were specifically selected from the extreme ends of the PFAS-exposure distribution. Even when accounting for measurement uncertainty, the low- and high-exposure groups remained well separated. Therefore, uncertainty in PFAS measurements did not affect participant classification or subsequent correlation analyses.

### 2.6. Metabolomics Analysis

The data acquisition platforms were previously optimized for both polar and apolar fractions [35,36]. Details on the LC and quadrupole time-of-flight (QToF) parameters are provided in Supplementary Materials Table S1. Briefly, the polar fraction was analyzed using an Agilent 1290 Infinity UPLC system coupled to an Agilent 6530 QToF-HRMS equipped with an Agilent Jet Stream ESI source (Agilent Technologies, Santa Clara, CA, USA). In ESI (+), separation was achieved on an iHILIC-Fusion column (100 × 2.1 mm, 1.8 μm, zwitterionic, charge-modulated amide, silica-based, Achrom Nv, Zulte, Belgium) with H_2_O/MeOH (9/1, *v*/*v*) containing 10 mM NH_4_COOH and 0.1% (*v*/*v*) HCOOH as mobile phase A (MPA) and MeCN as mobile phase B (MPB). In ESI (−), an iHILIC-Fusion(P) column (100 × 2.1 mm, 5 μm, zwitterionic, charge-modulated amide, polymer-based, HILICON AB) was used with 2 mM CH_3_COONH_4_ and 2 mM (NH_4_)_2_CO_3_ in H_2_O as MPA and MeCN/MeOH (9/1, *v*/*v*) as MPB.

The apolar fraction was analyzed using an Agilent 1290 Infinity II LC system coupled to an Agilent 6560 drift tube-ion mobility (DTIM)-QToF-HRMS equipped with a Dual Jet Stream ESI source. Separation was performed on an ACQUITY UPLC BEH C18 column (50 × 2.1 mm, 1.7 μm, Waters Corporation, Milford, MA, USA) for both ESI (+) and ESI (−) modes. MPA consisted of 5 mM CH_3_COONH_4_ in H_2_O/MeCN (7/3, *v*/*v*), while MPB was 5 mM CH_3_COONH_4_ in H_2_O/MeCN/IPA (2/10/88, *v*/*v*/*v*). For the ESI (+) mode, 0.1% (*v*/*v*) CH_3_COOH was added to the aqueous fraction of both MPA and MPB. Throughout the analytical run, a calibrant solution containing purine (*m*/*z* 121.0508 for ESI (+) and *m*/*z* 119.0363 for ESI (−)) and hexakis (1H, 1H, 3H-tetrafluoropropoxy) phosphazine (*m*/*z* 922.0097 for ESI(+) and *m*/*z* 966.0007 for ESI (−)) was continuously introduced via an auxiliary isocratic pump to maintain mass axis calibration during the analysis of both polar and apolar fractions.

Prior to every analytical run, a system suitability sample containing a minimum of 10 reference standards per analytical platform was injected to verify compliance with predefined criteria: peak height (≥5000 counts, RSD < 30%), mass error (≤10 ppm), and retention time (RT) deviation (≤0.2 min). The samples were randomized prior to injection, and the data were acquired using full scan (MS1) in profile mode using a 2 GHz extended dynamic range. The QC pooled samples were injected at least seven times at regular intervals [34] throughout the analytical sequence. Data-dependent acquisition (DDA) was initially performed on the QC pooled samples during system conditioning. For the apolar fractions, DDA with iterative exclusion was additionally applied to expand the scope of the lipidome covered, both increasing the total number and diversity of lipids annotated [37]. For the apolar fraction, 4 different pooled QC samples were analyzed, resulting in a total of 120 DDA injections. For each pooled QC sample, 9 auto-MS/MS and 6 auto-MS/MS with iterative exclusion injections were acquired, in both positive and negative ESI modes. For the polar fraction, 4 different pooled QC samples were analyzed, resulting in a total of 72 DDA injections, with 9 auto-MS/MS injections per QC in both ESI modes. Details on the MS parameters can be found in Supplementary Materials Table S1.

### 2.7. Statistical Analysis

Individual PFAS concentrations were measured in serum within a validated analytical range of 0.2–30 ng/mL, extending up to 480 ng/mL.

Imputation was conducted on a per-compound basis using a distribution-based single random imputation framework. This was applied only if at least 30% of measurements were above the limit of detection (LOD) or limit of quantification (LOQ). Concentrations were assumed to follow a lognormal distribution and to be left-censored. For each compound, the mean and standard deviation of the natural logarithm of observed values above LOD/LOQ were calculated. A censored lognormal distribution was then fitted using maximum likelihood estimation and truncated to analytically plausible bounds. For censored values below the LOD or LOQ, random values were drawn from the truncated distribution between 0 and the respective LOD or LOQ. For values reported as between LOD and LOQ, the sampling range was defined between LOD and LOQ. Compounds with fewer than 30% of measurements above LOD/LOQ were not subjected to random imputation, and censored values were treated as missing for that compound. No multiple-imputation framework was used.

The total PFAS serum concentration was calculated for each participant as the sum of the individual PFAS concentrations measured in their serum sample. For PFAS present as both linear and branched isomers, including PFOA, PFHxS and PFOS, total concentrations were quantified using the corresponding linear reference standards. For the total PFAS calculation, concentrations below the LOQ were replaced by half the LOQ on a per-compound basis prior to summation.

All participants included in the analysis had available PFAS measurements. Therefore, no missing values were present in the analytical dataset. Censored values were treated as described above and were considered to result from concentrations below analytical detection limits rather than from non-random missingness, consistent with established biomonitoring practice and HBM4EU guidelines [38].

Differences between the high- and low-exposure groups were assessed using the χ^2^ test for sex and the Mann–Whitney U-test for age, BMI, and total PFAS serum concentration. Effect sizes were estimated using the Phi coefficient for sex and Cliff’s delta test (95% CI) for age and BMI.

For the untargeted metabolomics, the raw LC-HRMS data acquired in the Agilent .d format were converted to the open-source .mzML format using MSConvert (ProteoWizard, v. 3.0.19317) [39]. Subsequently, these files were processed in MZmine (v. 4.3.0) [40,41]. The specific parameters utilized in MSConvert and MZmine are outlined in Supplementary Materials Table S2. The resulting data matrix was imported into MS-FLO for further deisotoping and duplicate removal [42]. The MS-FLO processing parameters are detailed in Supplementary Materials Table S3. Data quality was evaluated by plotting the relative standard deviation (RSD) of each feature intensity for each sample group. Subsequently, drift correction was applied using a cubic spline approach implemented in the Notame R-package (R v. 4.1.2) [43]. To ensure high-quality features, multiple filtration criteria were applied. Features with a maximum intensity < 10 times the mean extraction blank intensity were removed. In addition, a feature was required to be detected in at least 75% of any sample group and in at least 70% of QC pooled samples. Only features exhibiting an RSD < 30% for intensity and a D-ratio < 40% were retained. After these filtering steps (including QC filtering), multivariate statistical analyses were performed on pretreated data that underwent missing value imputation using random forest (RF, missForest R-package, R v. 4.1.2), with QC samples removed prior to imputation to ensure that missing values were estimated based on patterns in the biological data [43], log-transformation of intensity values [43,44], probabilistic quotient normalization (PQN) using the QC pooled median intensity, and Pareto scaling [43,45,46,47,48,49,50]. QC samples were imputed separately using the median value of each feature to preserve technical consistency. Outliers were identified and excluded based on the number of detected features per sample relative to other samples from the same group (samples outside Q1 − 1.5 × IQR or Q3 + 1.5 × IQR), the principal component analysis (PCA) plots [51], and deviations in IS detection. The predefined acceptance criteria for IS deviation included a peak height exceeding 5000 counts without signal saturation, a mass accuracy < 10 ppm, an RT deviation < 0.5 min for HILIC, and <0.2 min for RPLC. Outliers were set as missing to avoid biasing subsequent imputation. Both univariate and multivariate statistical approaches were used separately to select features that distinguish the low- and high-exposure groups for further annotation. Covariate adjustment was not applied during feature selection, as the goal was to capture overall differences between groups. Univariate statistical analyses were performed before data treatment. The normality of each feature was evaluated individually using the Shapiro–Wilk test based on its intensity values. Based on the statistical significance (*p*-value < 0.05) of the Shapiro–Wilk test, a Mann–Whitney U-test or a student *t*-test with Benjamini–Hochberg (BH) correction was performed [52,53], and fold changes (FC) were calculated and log_2_-transformed for visualization. For univariate analysis, features with a false discovery rate (FDR) < 0.05 and an FC > 1.2 or <0.8 relative to the low-exposure group were considered significant. Multivariate analyses were also performed to take into consideration associations within the broader network, facilitating biological interpretation during pathway analysis of selected interesting features. The multivariate workflow included a partial least squares-discriminant analysis (PLS-DA) with 10-fold cross-validation and a binary RF classifier [54]. The PLS-DA performance was assessed using a permutation test of the response variable (*n* = 1000) and by evaluating the area under the curve (AUC), R^2^ and Q^2^ metrics, whereas the RF model was evaluated based on the AUC. Interesting features prioritized for further annotation were selected based on their variable importance in projection score > 1 in the PLS-DA model and their mean decrease in accuracy in the RF model. Features selected by either the univariate and/or multivariate approaches were retained.

To better understand the biological relevance of the altered lipids, lipid pathway enrichment was performed using LIPEA [23], with annotated lipids mapped to KEGG identifiers and the predefined human lipidome as background. Enrichment was assessed using Fisher’s exact test with BH correction [26]. Pathways with FDR < 0.05 were considered significant. Metabolite pathway analysis was conducted in MetaboAnalyst (v. 6.0) [24] using a reference metabolome of all platform-detectable metabolites. Fisher’s exact test and relative betweenness centrality were used for enrichment and topology analysis, respectively. KEGG pathways for *Homo sapiens* (downloaded December 2024) served as the pathway library. Pathways with FDR < 0.05 and a topological impact score higher than 0.2 were considered significant. No covariate adjustment was applied during pathway analyses, as the aim was to capture overall pathway perturbations associated with high vs. low PFAS exposure.

Spearman’s rank correlation coefficient was computed using MetaboAnalyst (v. 6.0) between the total PFAS concentration and intensities of altered metabolites in high-exposure compared to low-exposure groups in order to identify significant associations. Correlations were interpreted based on the absolute value of the correlation coefficient (r), with the following categories: very weak (|r| < 0.2), weak (|r| = 0.2–0.39), moderate (|r| = 0.40–0.59), and strong (|r| ≥ 0.60), according to commonly used guidelines for interpreting correlation strength [55]. To evaluate the potential influence of covariates (age, sex, BMI, and blood collection time), sensitivity analyses were performed in R (v. 4.1.2) using individual linear regression models on log-transformed metabolite intensities. For metabolites significantly correlated with total PFAS concentration, associations were evaluated in unadjusted (PFAS only) and covariate-adjusted models, with total PFAS concentration as the predictor of interest. Variance inflation factors (VIFs) were calculated for all covariates to evaluate multicollinearity.

Classical univariate Receiver Operating Characteristic (ROC) curve analysis was also performed in MetaboAnalyst (v. 6.0) for individual metabolites, generating ROC curves, AUC, and 95% confidence intervals (calculated using 500 bootstraps). Metabolites with *p*-value < 0.05 in the ROC analysis were considered significant and were used to exploratorily assess their discriminatory performance, rather than as a pre-specified biomarker threshold. The intensities of the altered metabolites were log-transformed, PQN-normalized, and Pareto-scaled before constructing a multivariate panel. Out of the 38 annotated metabolites, metabolites were grouped into five distinct clusters using hierarchical clustering, which organizes metabolites based on similarity in their patterns across samples. Feature selection was performed using PLS-DA ranking across multiple cross-validation iterations. From the top 12 ranked features, based on their selection frequency, the metabolites exhibiting the highest individual discriminatory performance (as measured by AUC) within each cluster were selected for inclusion in the selected panel. One representative metabolite from each cluster was selected to reduce redundancy while capturing the main metabolic signals distinguishing low- and high-exposure groups. The performance of the selected metabolite panel was evaluated using a linear Support Vector Machine classification model. Monte-Carlo cross-validation with balanced sub-sampling was employed to reduce the risk of overfitting: in each iteration, two-thirds of samples were used for training and one-third for testing. This process was repeated multiple times to provide robust estimates of model performance and feature importance. Class-label permutation tests (*n* = 1000) were also performed to ensure that the observed predictive performance was unlikely to be due to chance (*p*-value ˂ 0.05). Model performance was additionally summarized using the mean predictive accuracy across 100 validation runs. To evaluate the potential influence of covariates (age, sex, BMI, and blood collection time) on the selected panel, sensitivity analyses were performed in R (v. 4.1.2) using log-transformed, PQN-normalized, and Pareto-scaled metabolite intensities. Linear regression models were fitted for individual metabolites and for the combined five-metabolite panel, comparing unadjusted and covariate-adjusted associations with total PFAS concentration. Logistic regression models were also used to assess the ability of individual metabolites and the panel to discriminate high vs. low PFAS exposure, both unadjusted and adjusted for covariates. VIFs were calculated for all covariates to assess multicollinearity.

Effect sizes for individual metabolites were calculated using Cohen’s d to quantify the magnitude of PFAS-associated differences between high- and low-exposure groups. Statistical power for each metabolite was estimated based on the calculated Cohen’s d and the cohort size (*n* = 41 per group), assuming a two-sided independent *t*-test with α = 0.05. The non-centrality parameter (δ) for each metabolite was calculated as δ = d × √(n_1_n_2_/(n_1_ + n_2_)), where n_1_ and n_2_ are the sample sizes of the two groups. Power was then derived from the non-central t-distribution using the calculated δ and the degrees of freedom (df = n_1_ + n_2_ − 2). This approach enabled assessment of the reliability of the selected panel metabolites and provided context for interpreting metabolites with smaller effect sizes.

### 2.8. Metabolite Annotation

The pooled QC MS/MS files were used both for metabolite annotation and for confirmation of lipid class assignments, following established untargeted metabolomics practices [34]. A tandem mass spectral library (MS/MS) search was carried out to annotate metabolites in both polar and apolar fractions [56]. The All Public MS/MS library (v. 15), the NIST library (v. 17), and MassBank of North America (https://mona.fiehnlab.ucdavis.edu/, accessed on 4 January 2026) were used within MZmine (v. 4.3.0) [40,41]. The annotation confidence was enhanced by manual inspection of matched MS/MS spectra (±5 ppm precursor ions, ±10 ppm fragment ions, 4–15 diagnostic fragments), utilizing an in-house library (available in MassBankEU https://doi.org/10.5281/zenodo.8308157) to achieve higher confidence levels (CLs) when the standards were available, and rule-based fragmentation was applied for manual evaluation of annotated lipids [57,58,59]. When fragmentation spectra indicated mixtures of molecular species, these were reported collectively under their corresponding bulk lipid. Polar metabolites were reported according to the RefMet [60] shorthand notation system and according to the annotation confidence system of Schymanski et al. [61]. Features that could be annotated with a CL of 3 or higher were reported. Lipids were reported following the nomenclature rules of Liebisch et al. [62] (LIPID MAPS) using shorthand lipid notation.

## 3. Results

### 3.1. Cohort Characteristics

The main characteristics of the cohort subgroups—including biological sex, age, BMI, and total PFAS serum concentration—are summarized in Table 1. The low- and high-exposure groups (*n* = 41 each) were comparable in sex distribution, BMI, and age, with age showing a non-significant trend toward being higher in the high-exposure group (*p* = 0.06). Effect size analyses further supported these observations: the difference in age was small and uncertain (Cliff’s delta = −0.239, 95% CI [−0.462, 0.013]), BMI distributions were nearly identical between the exposure groups (Cliff’s delta = 0.093, 95% CI [−0.163, 0.337]), and the difference in sex distribution was small (Phi coefficient = 0.182). Taken together, these findings suggest that age, BMI, and sex are unlikely to substantially confound potential associations. Serum total PFAS concentrations were substantially higher in the high-exposure group than in the low-exposure group (median 162.0 vs. 7.2 ng/mL; *p* < 0.01). The PFAS profile was similar across exposure groups and dominated by three compounds (PFOS, PFOA, and PFHxS), with PFOS present in much higher concentrations than PFOA (about 5–50-times higher) and PFHxS (about 10–20-times higher). As such, PFOS dominate the PFAS profile, accounting for 85–95% of the total PFAS. Other PFAS (e.g., PFNA, PFDA, PFHpS) were detected at lower concentrations in both groups and did not alter this overall pattern.

Among women, 55% reported a history of past breastfeeding, whereas only 7% were currently breastfeeding at the time of sampling. No significant differences in breastfeeding status were observed between exposure groups (χ^2^ test, *p*-value > 0.05). Given the low prevalence of current breastfeeding, any potential confounding effect is expected to be minimal. Data on some potential confounders for metabolomics, such as smoking, alcohol consumption, comorbidities, and medication use, were not available for this cohort. Nevertheless, the two exposure groups did not differ significantly in financial situation based on income or household education level (χ^2^ tests, *p*-value > 0.05 for both). The difference in blood collection times between the high- and low-exposure groups was minimal and uncertain (mean difference = 0.45 h, 95% CI [−0.83, 1.72], *t*-test *p* = 0.488, Cohen’s d = 0.15), indicating that collection time is unlikely to substantially confound group comparisons.

### 3.2. Data Quality Management

Following LC-HRMS data acquisition, the extracted ion chromatograms of the ISs were visually inspected to identify samples with outlier IS peak intensities. The RSD of the molecular features was calculated per group. For untargeted metabolomics using LC-HRMS, RSD values ≤ 30% are generally considered acceptable [63,64]. The median RSD (mRSD) of the pooled QC samples was determined to assess the analytical precision of the dataset. Across all platforms, mRSD values for QC samples were below 15%, indicating a reliable analytical platform [64]. A summary of mRSD values for each polarity, platform and exposure group is provided in Supplementary Materials Table S4. The higher mRSD observed in the serum sample group compared to the QC samples is attributed to biological variability and sample preparation differences. For instance, in the apolar ESI (+) fraction, the mRSD increased from 11% to 29%. Similarly, the polar ESI (+) mRSD increased from 14% to 32%.

**Figure 1:**
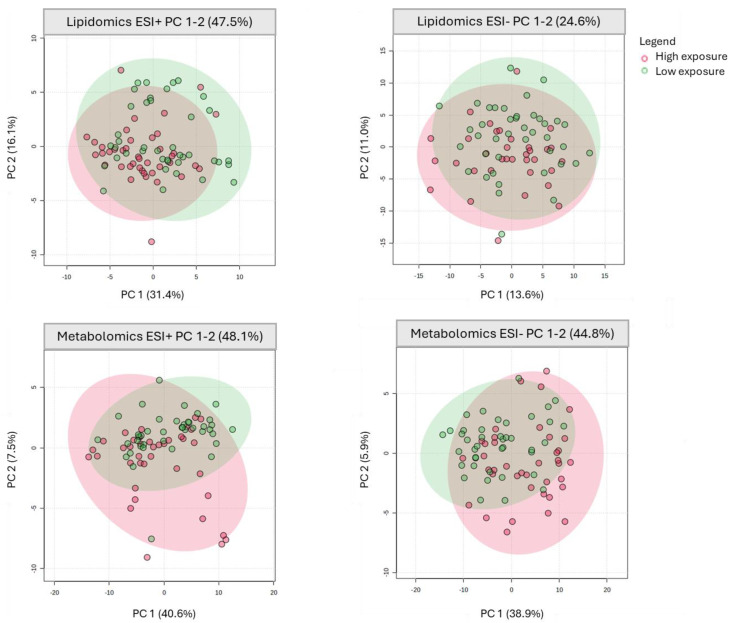
Principal component analysis plots of serum from PFAS-exposure groups analyzed in different ionization modes (ESI (+) and ESI (−)). Lipidomics plots correspond to the apolar fraction, whereas metabolomics plots correspond to the polar fraction. A partial separation is observed between the high-exposure group (green) and the low-exposure group (red), indicating some inter-group variability. Negative values on the PCA axes are shown as hyphens due to automatic rendering by MetaboAnalyst. PCA plots with QC samples are provided in Supplementary Materials Figure S2.

An unsupervised PCA was first carried out to explore correlations between variables and to obtain an overview of the metabolomics dataset prior to supervised modeling [65]. PC1 and PC2 together explained 24.6–48.1% of the total variation, depending on the analytical platform, thus capturing important sources of variability (Figure 1). The PCA score plots (Supplementary Materials Figure S2) show tight clustering of QC samples, demonstrating good instrument reproducibility [66]. The sample groups exhibited partial separation, indicating some inter-group variability.

PLS-DA and RF models were constructed to select features of interest for subsequent annotation. Given the risk of overfitting in PLS-DA, model validation was performed using 10-fold cross-validation along with a permutation test of the response variable (*n* = 1000) (Table 2). PLS-DA evaluation metrics indicated modest predictive ability (AUC ≥ 0.70, R^2^ ≥ 0.30, Q^2^ ≥ 0.14), with permutation tests showing statistical significance for the apolar platforms (*p* < 0.05), suggesting that the models captured PFAS exposure-associated metabolic patterns without overfitting. In contrast, PLS-DA models for the polar platforms were non-significant in permutation tests (*p* > 0.05) and were therefore not used for multivariate feature selection. RF models demonstrated moderate classification performance (AUC ≈ 0.7) across all platforms.

### 3.3. Metabolic Fingerprint of PFAS Exposure in Serum of a Flemish Population Residing near a PFAS Production Facility

The features selected based on the univariate and/or multivariate statistical approaches and showing distinct alteration between the two exposure groups were kept for annotation. Supplementary Materials Tables S5 and S6 list the annotated metabolites together with their corresponding retention time, *m*/*z*, and ionization species. After statistical analysis, 38 features were annotated, 5% were polar metabolites, and 95% were lipids. For polar metabolites, annotations were performed using an in-house library, the All Public MS/MS library (v. 15) within MZmine, NIST (v. 17), and MoNA. The in-house library enabled the confirmation of L-aspartic acid at a CL of 1. Propionylcarnitine (CAR 3:0) was annotated at a CL of 2a using NIST and MoNA. For the apolar fraction, annotations were carried out with an in-house library and the All Public MS/MS library (v. 15) in MZmine.

**Figure 2:**
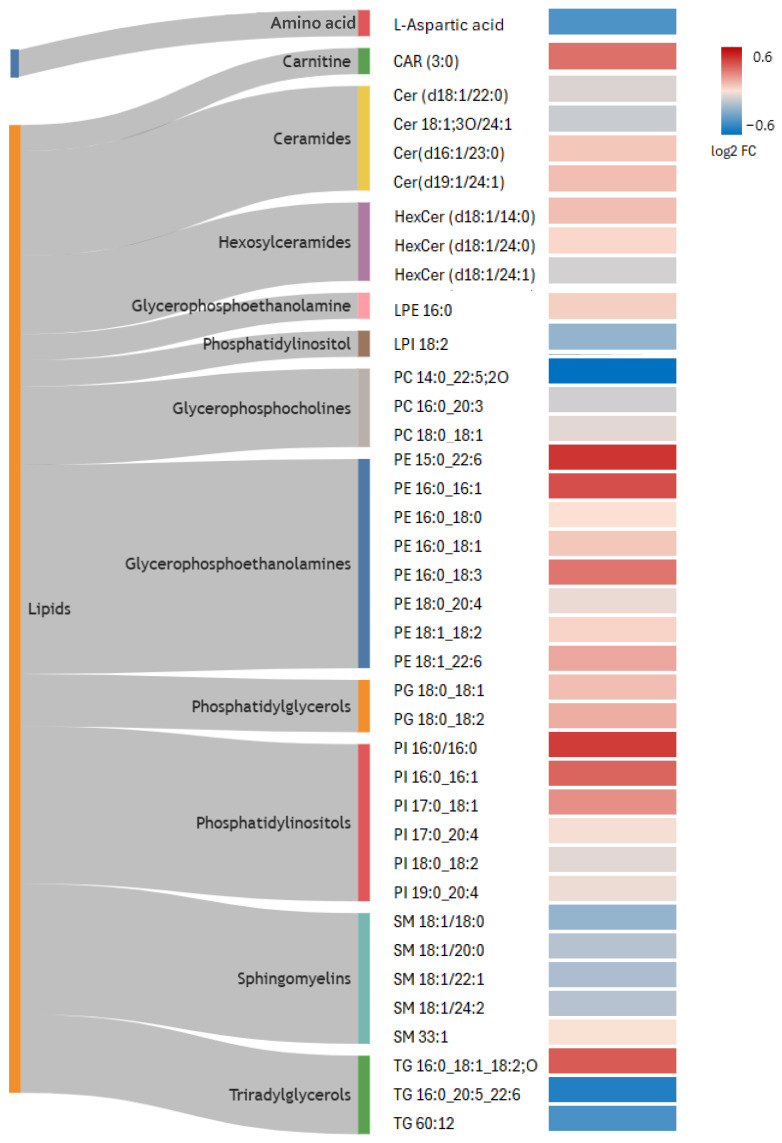
Heatmap of the annotated metabolites showing transformed fold changes, highlighting the effects of PFAS exposure on serum metabolite profile. Log_2_ fold changes were derived from peak intensities and represent the ratio of the high-PFAS-exposure group relative to the low-PFAS-exposure group, with the low-exposure group serving as the reference. A Sankey diagram was used to assign lipid classes following the LIPID MAPS classification [62]. FC: fold change, CAR: carnitine, Cer: ceramide, HexCer: hexosylceramide, LPE: lysophosphatidylethanolamine, LPI: lysophosphatidylinositol, PG: phosphatidylglycerol, PI: phosphatidylinositol, SM: sphingomyelin, PC: phosphatidylcholine, PE: phosphatidylethanolamine, TG: triglyceride.

The impact of PFAS exposure on each annotated metabolite is illustrated using a heatmap of the log_2_-transformed FC alongside a Sankey diagram for metabolite classification (Figure 2). Within the polar fraction, L-aspartic acid was downregulated in serum in the high-exposure group compared with the low-exposure group, and CAR 3:0 was upregulated in the high-exposure group. In the apolar fraction, ceramides (Cer) containing a d18:1 backbone showed a slight decrease in the high-exposure group, while d16 and d19 backbone ceramides were increased. Hexosylceramides (HexCer) showed a subtle change with a tendency toward increased saturated species in the high-exposure group, while unsaturated species remained relatively stable. Phosphatidylcholines (PC) were generally stable or decreased. Among the annotated lipids, phosphatidylethanolamines (PE) including lysophosphatidylethanolamines (LPE) constituted the largest proportion and, overall, tended to increase in the high-exposure group, with PE 15:0_22:6 showing the highest FC. Two phosphatidylglycerol (PG) species were annotated, both showing upregulation with high PFAS exposure. Among phosphatidylinositols (PIs), lysophosphatidylinositol (LPI 18:2) showed an opposite trend, whereas other PIs increased. Similarly, sphingomyelins (SMs) were mostly decreased, except for SM 33:1. Triacylglycerols (TGs) displayed divergent responses, with TG 16:0_18:1_18:2;O increased and the other TGs decreased by high PFAS exposure.

### 3.4. Metabolic Pathway Analysis

Pathway enrichment analysis of the selected metabolites identified eight significantly enriched lipid-associated pathways, suggesting overrepresentation of exposure-associated lipid changes in these metabolic routes (Supplementary Materials Table S7). These pathways mainly showed perturbations in glycerophospholipid metabolism, sphingolipid metabolism, sphingolipid signaling, retrograde endocannabinoid signaling, glycosylphosphatidylinositol (GPI)-anchor biosynthesis, autophagy, and necroptosis. When applying pathway topology analysis in MetaboAnalyst, only two of these pathways (glycerophospholipid metabolism and sphingolipid metabolism) exhibited moderate impact (Table 3), indicating that alterations occurred at topologically central nodes within the metabolic network rather than broadly across all enriched pathways. The use of combined enrichment and topology approaches thus highlights not only statistically overrepresented pathways, but also those with potentially greater biological relevance due to their structural role in metabolism. However, because the untargeted serum metabolomics and lipidomics captured only a subset of circulating lipids and pathway mapping/topology (impact) scores are computational predictions, these pathway annotations should be interpreted as exploratory and hypothesis-generating rather than evidence of definitive in vivo pathway alteration.

**Figure 3:**
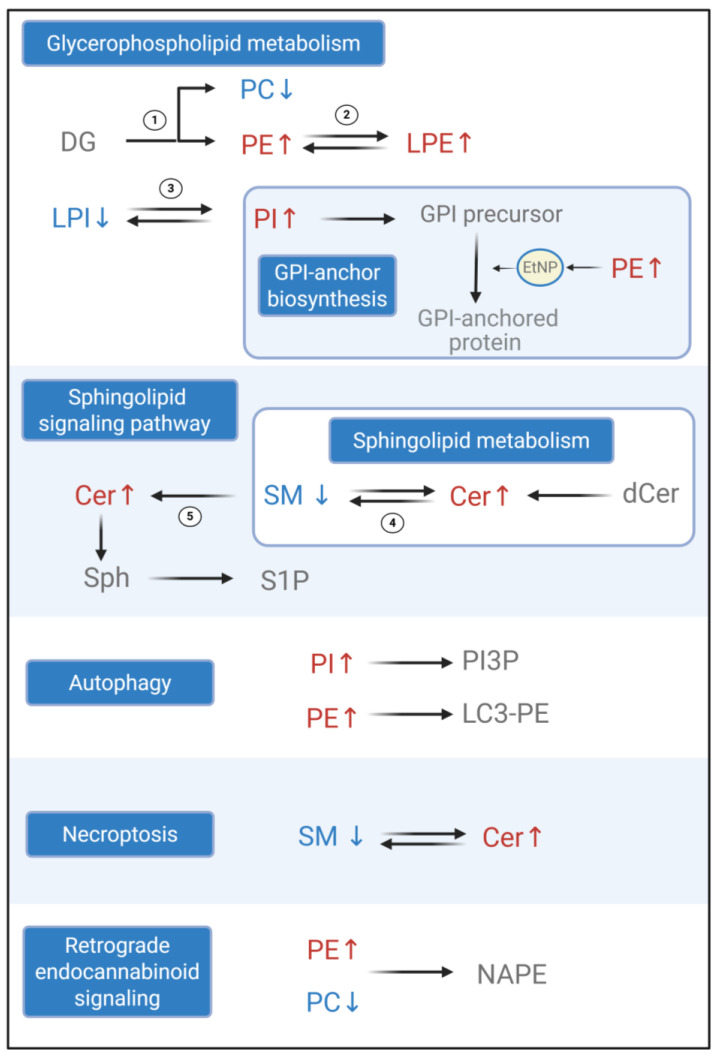
Metabolic pathways enriched in the high-PFAS-exposure group. Names and arrows in blue correspond to downregulation; names and arrows in red correspond to upregulation. Black arrows indicate the reactions discussed in Section 3.4. Numbered labels (1–5) distinguish multiple reaction steps within the same pathway. This figure provides a conceptual, pathway-level overview of lipid alterations between PFAS-exposure groups and does not represent quantitatively measured metabolic fluxes. DG: diglyceride, PC: phosphatidylcholine, PE: phosphatidylethanolamine, LPE: lysophosphatidylethanolamine, LPI: lysophosphatidylinositol, PI: phosphatidylinositol, GPI: Glycosylphosphatidylinositol, EtNP: ethanolamine phosphate, SM: sphingomyelin, Cer: ceramide, Sph: sphingosine, S1P: sphingosine-1-phosphate, PI3P: phosphatidylinositol 3-phosphate, LC3-PE: LC3–phosphatidylethanolamine conjugate (LC3-II), NAPE: N-acyl-phosphatidylethanolamine. Created in BioRender. Sher, H. (2026) https://BioRender.com/a0bc1yl, accessed on 4 January 2026.

Glycerophospholipid metabolism was perturbed when comparing the different PFAS-exposure groups. PC and PE are synthesized de novo via the Kennedy pathway, in which diacylglycerol (DG) is converted to PC through the CDP-choline branch and to PE through the CDP-ethanolamine branch. Accordingly, the observed decrease in PC, together with an increase in PE, could be consistent with a redistribution of pathway flux favoring PE production over PC synthesis [69] (Figure 3, reaction 1). Notably, LPE increased alongside PE, which could be consistent with altered PE turnover through phospholipid remodeling pathways. Lysophospholipids could be generated via phospholipase A–mediated deacylation of diacyl phospholipids (including PE → LPE) and subsequently reacylated by lysophospholipid acyltransferases as part of the Lands’ cycle [70] (Figure 3, reaction 2). PI–LPI remodeling could also be implicated, and the increase in PI with a slight decrease in LPI may indicate a relative bias toward maintaining diacyl-PI in circulation (Figure 3, reaction 3). In GPI-anchor biosynthesis, a pathway linked to glycerophospholipid metabolism, PI serves as the lipid backbone on which the anchor is assembled, whereas PE provides ethanolamine phosphate (EtNP) groups required for GPI maturation and protein attachment [71].

Altered levels of ceramides and SMs were also observed between both PFAS-exposure groups, potentially indicating changes in sphingolipid metabolism and signaling pathways. Ceramides, key intermediates in sphingolipid metabolism, were found altered alongside SMs when comparing the high- and low-PFAS-exposure groups. Ceramides are synthesized in the endoplasmic reticulum and transported to the Golgi apparatus, where they are converted to sphingomyelin, and they can also be generated through the hydrolysis of SM (Figure 3, reaction 4) [72]. Ceramides are further converted into signaling intermediates and complex sphingolipids such as SMs. These downstream signaling intermediates can mediate diverse cellular processes, including pro-apoptotic and pro-growth responses. Ceramides are converted to sphingosine by ceramidases and subsequently phosphorylated to generate sphingosine-1-phosphate (S1P), a potent lipid signaling mediator associated with cellular survival and the suppression of apoptosis (Figure 3, reaction 5) [67]. The upregulation of ceramides may suggest a disruption of the ceramide–S1P balance, potentially leading to perturbations in diverse cellular processes.

Enrichment analysis linked elevated PI and PE in the high-PFAS-exposure group to autophagy-associated pathway annotation. In autophagy, PI serves as a substrate for phosphatidylinositol 3-phosphate (PI3P) in early phagophore formation, whereas PE is required for LC3–phosphatidylethanolamine conjugation (LC3-PE) during autophagosome membrane expansion [73,74]. The increase in ceramides and decrease in SMs aligned with necroptosis-associated pathway annotation. Sphingomyelin-to-ceramide conversion has been associated with regulated necrotic cell death processes, whereas the reverse reaction is generally linked to membrane homeostasis and pro-survival signaling [75]. Retrograde endocannabinoid signaling was highlighted based on altered PE and PC features. In this pathway, N-acyl-phosphatidylethanolamine (NAPE)—an immediate precursor of anandamide—can be formed via N-acylation of PE, typically using acyl chains donated from PC or other phospholipids [76,77].

### 3.5. Metabolites Association with PFAS Exposure

**Figure 4:**
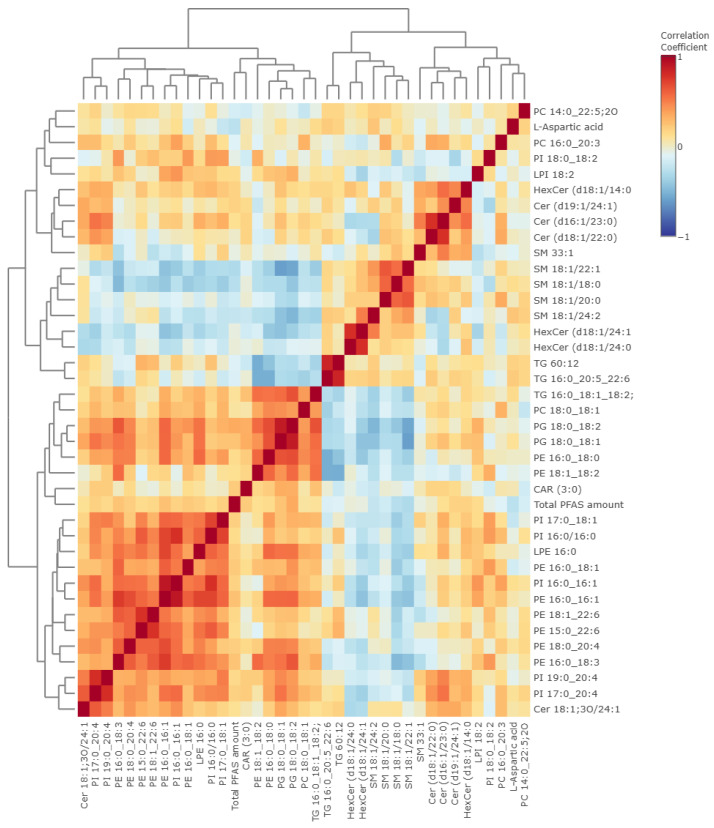
Spearman correlation heatmap among the 38 selected metabolites and the total PFAS serum concentration. The color of each cell reflects the correlation coefficient, with red indicating positive and blue indicating negative correlations.

The correlation between altered metabolites and total PFAS concentration was evaluated to identify metabolites associated with PFAS exposure. Spearman correlation analysis was performed for the 38 annotated metabolites (Figure 4).

In total, 8 of the 38 altered metabolites showed significant associations with the total PFAS serum concentration (Table 4). Among lipids, positive correlations were observed for several phospholipid species, including PE 16:0_16:1, PG 18:0_18:2, PG 18:0_18:1, and PE 16:0_18:3, indicating the higher level of these lipids in residents with a higher total PFAS serum concentration. In contrast, SMs (SM 18:1/24:2, SM 18:1/18:0, and SM 18:1/22:1), as well as L-aspartic acid, were inversely associated with the total PFAS serum concentration, indicating lower levels of these compounds in residents with higher PFAS exposure. These weak-to-strong correlations highlight the metabolites most closely associated with the total PFAS serum concentration.

Covariate adjustment was not applied in the correlation analysis. Sensitivity analyses, adjusting for age, sex, BMI, and blood collection time, showed that the direction and magnitude of PFAS effect estimates remained largely unchanged compared with unadjusted models (e.g., aspartic acid: β = −0.00088 unadjusted vs. −0.00065 adjusted, PE 16:0_16:1: β = 0.00034 vs. 0.00051). These findings suggest that the covariates had a limited impact on the observed associations.

To explore their ability to discriminate high- vs. low-exposure individuals, individual ROC analyses were performed for all 38 annotated metabolites (Supplementary Materials Table S8), regardless of their correlation with total PFAS. Among these, L-aspartic acid exhibited the highest individual discriminatory performance (AUC = 0.722, 95% CI [0.593–0.829]). Several SMs, including SM 18:1/22:1 (AUC = 0.673) and SM 18:1/18:0 (AUC = 0.654), demonstrated a moderate discriminatory performance. Other lipids such as PG 18:0_18:2 (AUC = 0.653), PI 17:0_18:1 (AUC = 0.619), HexCer 18:1/14:0 (AUC = 0.623), and SM 18:1/20:0 (AUC = 0.626) showed a modest performance. These results highlighted candidate metabolites with a statistically significant discriminatory performance in distinguishing PFAS-exposure groups.

**Figure 5:**
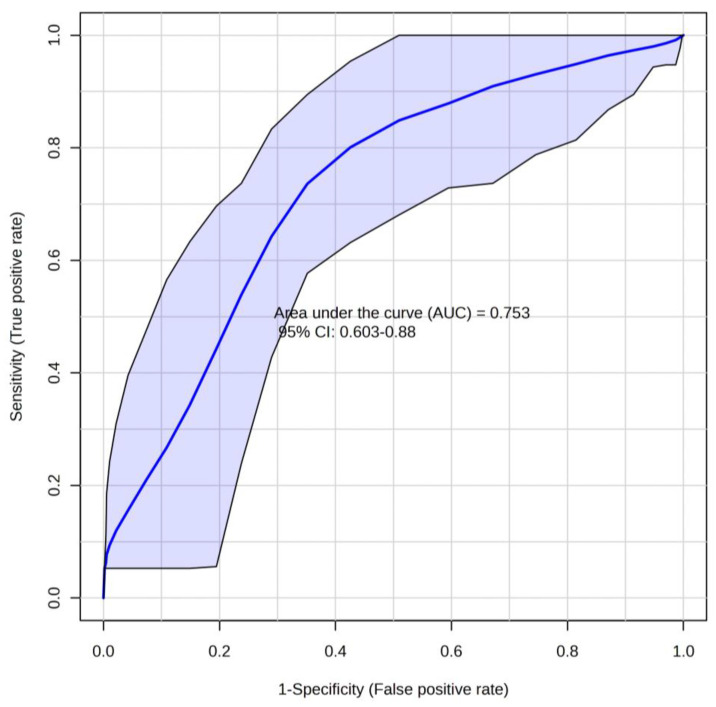
ROC curve obtained from an exploratory panel of 5 metabolites, illustrating their discriminatory performance between high- and low-PFAS-exposure residents. The 95% confidence interval (CI) is shown with a hyphen due to automatic rendering by MetaboAnalyst.

To integrate complementary information from individual metabolites, a multivariate panel was constructed. This approach does not aim to replace serum PFAS measurements for exposure classification, but rather to explore whether the metabolites in the selected panel collectively form a potential metabolic signature associated with PFAS exposure. The five compounds in the selected panel included L-aspartic acid, PG 18:0_18:2, PE 16:0_18:3, HexCer (d18:1/14:0), and TG 16:0_20:5_22:6. The panel showed moderate discriminatory ability between individuals with high PFAS levels from those with low levels, achieving an AUC of 0.753 (95% CI [0.603–0.880]) (Figure 5). Among the panel components, L-aspartic acid showed the highest individual discriminatory performance (AUC = 0.722), which was lower than that of the combined five-metabolite panel. This comparison supports the added value of multivariate integration over reliance on a single metabolite. Cross-validation confirmed stable model performance, with a mean predictive accuracy of 0.697 across 100 validation runs and correct classification of over 66% of samples. Class-label permutation test supported the statistical significance of the panel (*n* = 1000, *p* = 0.009). Boxplots showing the distribution of quantitative responses detected of each panel metabolite across exposure groups are presented in Supplementary Materials Figure S3. Key metrics, including individual AUC, FC, Cohen’s d, and correlation with total PFAS, are summarized in Supplementary Materials Table S9.

Sensitivity analyses were conducted using linear and logistic regression models to assess whether age, sex, BMI, and blood collection time influenced the observed associations. A comparison between the unadjusted models (PFAS only) and covariate-adjusted models showed that the direction and magnitude of the PFAS effect estimates remained consistent, indicating that these covariates had a minimal impact. Covariate adjustment was not applied in the exploratory panel analyses.

To further assess the reliability of the selected panel, effect sizes (Cohen’s d) (Supplementary Materials Figure S4) and estimated statistical power were calculated for all 38 altered metabolites. The five panel metabolites, including L-aspartic acid (d = −0.84, *p* = 0.001, power ~95%), PG 18:0_18:2 (d = 0.50, *p* < 0.001, power ~62%), PE 16:0_18:3 (d = 0.40, *p* = 0.093, power ~48%), HexCer (d18:1/14:0) (d = 0.48, *p* = 0.040, power ~59%), and TG 16:0_20:5_22:6 (d = −0.43, *p* = 0.074, power ~53%), demonstrated moderate-to-strong effect sizes and sufficient statistical power to reliably capture exposure-associated alterations within the cohort. Additional metabolites exhibited smaller effect sizes.

## 4. Discussion

A key methodological consideration in this study is the selection of participants from the extremes of PFAS exposure, which maximizes contrast and makes it easier to detect differences in metabolites. However, because participants with intermediate PFAS exposure were not included, this may overestimate the observed effect sizes and the findings may not generalize to individuals with intermediate exposure levels [78,79]. Consequently, the reported differences in metabolites should be interpreted as contrasts between low- and high-exposure groups, rather than as reflecting dose–response relationships across the entire exposure spectrum.

The total PFAS serum concentration in the high-exposure group (median total PFAS = 162.0 ng/mL) was substantially higher than that reported for the general Belgian population. In adults from Wallonia (Liège Province), the median serum concentrations of individual PFAS were approximately PFOS 4.3 ng/mL, PFOA 1.9 ng/mL, PFHxS 0.54 ng/mL, and PFNA 0.59 ng/mL, with the sum of all measured PFAS in adults remaining only a few ng/mL [80]. Similarly, in the Flemish Environment and Health Study (FLEHS), median adult concentrations were reported as PFOS 10.1 ng/mL, PFOA 3.2 ng/mL, PFHxS 1.6 ng/mL, and PFNA 0.9 ng/mL [81], with the sum of measured PFAS still below the 162 ng/mL observed in the high-exposure group of this cohort. This pronounced difference indicates that PFAS accumulation in this cohort is substantially greater than the background exposure levels reported for the Belgian general population.

Although PFOS, PFOA, and PFHxS dominated the PFAS profile, the composition was similar across participants with high and low exposure, suggesting that differences in compound dominance are unlikely to have a substantial influence on the observed metabolic associations.

Pathway enrichment and topology analysis indicated that lipid-centered pathways were the primary targets of PFAS-associated variation, with the strongest signals mapping to glycerophospholipid and sphingolipid metabolism. This pattern is consistent with prior human metabolome-wide association studies and reviews, which repeatedly identify glycerophospholipids and sphingolipids among the most reproducibly perturbed biochemical domains in relation to circulating PFAS [21,23,82]. Notably, PFAS-associated pathway enrichment for glycerophospholipid metabolism has been reported in human studies linking PFAS to lipid traits (e.g., LDL-related pathways), further supporting the relevance of this pathway to systemic PFAS-associated metabolic phenotypes [83]. Evidence from mouse liver studies and human hepatocytes experiments also indicates that PFAS can disrupt systemic lipid homeostasis by shifting glycerophospholipids such as PC, PE, and PI. PE upregulation has been observed consistently in mouse liver, human hepatocytes, and in the serum profiles reported in this study. These glycerophospholipid changes are accompanied by alterations in pathways linked to glycerophospholipid metabolism, supporting the biological relevance of the pathways observed in this study [84,85].

Ceramides and SMs are important components in sphingolipid metabolism and signaling. Consistent with our study, occupationally relevant PFOA exposure in mice has been reported to decrease hepatic SM and increase ceramide abundance, accompanied by the upregulation of sphingomyelinase, the enzyme responsible for converting sphingomyelin to ceramide [86]. Moreover, significant associations between sphingolipids and the linear isomers of PFOA and PFOS have been reported in human serum. Although SMs decreased in our study, indicating a different direction of regulation, the pronounced alterations observed for these major PFAS among 3647 participants suggest that PFAS exposure can perturb normal sphingolipid metabolism [87].

Enrichment of pathways connected to autophagy, supported by the observed upregulation of PE and PI, may reflect systemic metabolic responses rather than direct tissue-level autophagy and could be plausible when considering these metabolites as multifunctional hubs rather than isolated pathway components [88]. Autophagy initiation requires PI3P-generating activity, whereby PI serves as the lipid precursor that is phosphorylated to form phosphatidylinositol-3-phosphate (PI3P), and autophagosome biogenesis depends on PE-dependent lipidation of ATG8 family proteins (LC3/GABARAP), linking autophagy to PI-derived phosphoinositide and PE metabolism [73,74]. Consistent with this mechanistic framework, PFOA exposure has been reported to increase LC3-II (LC3-PE), an autophagy marker, in both mouse liver and hepatocyte-derived cells, providing experimental support for the involvement of autophagy-related processes under PFAS exposure [89].

Enrichment of GPI-anchor biosynthesis in serum may suggest PFAS-associated perturbations in membrane-anchoring processes and cell-surface organization. In a liver metabolomics study of NAFLD, GPI-anchor biosynthesis appeared among the pathways identified by overrepresentation analysis in relation to total PFAS exposure, although it was not a dominant pathway signal [90]. Complementary in vivo findings in zebrafish showed a trend toward altered GPI-anchor biosynthesis in male adult liver following developmental PFBS exposure, supporting the plausibility of PFAS sensitivity in this pathway across species [91].

Decreased SMs and increased ceramide in serum were also mapped to the enrichment of necroptosis, which may reflect systemic sphingolipid stress signatures associated with PFAS exposure rather than direct tissue-level necroptosis. An experimental study suggested that PFDA exposure can trigger necroptosis in granulosa cells and is accompanied by ovarian effects in mice [92]. In addition, PFOA has been reported to induce necrosis/necroptosis-like cell death in mouse-derived cells, with rescue by a necroptosis inhibitor [93]. In an in vitro study using HepaRG (human hepatocyte-like) cells exposed to legacy and alternative PFAS, pathway enrichment of the PFAS-altered lipid features identified necroptosis as an enriched pathway, with ceramides being the lipid class contributing to this signal, consistent with the Cer-related pattern observed in our serum data [94].

Retrograde endocannabinoid signaling was highlighted by increased PE and decreased PC in the high-PFAS-exposure group. A lipidomics study in human hepatocyte-like cells (HepaRG) has shown that PFAS (including PFOA/PFOS) can alter NAE levels and major phospholipid classes, supporting the idea that PFAS exposure may influence lipid networks connected to endocannabinoid signaling [94].

Additionally, a panel of five metabolites was constructed to reinforce the observed metabolic alterations associated with the PFAS-exposure groups. Some metabolites exhibited strong discriminatory performance and strong correlation with PFAS levels, while others showed weaker associations. Among the panel components, L-aspartic acid, with AUC 0.722, demonstrated the highest discriminatory power between PFAS-exposure groups, further supported by a large effect size (Cohen’s d = −0.84)). Although the correlation with PFAS levels was modest (r = −0.27), it was statistically significant, indicating an inverse association between this metabolite and PFAS exposure while suggesting that additional factors may also contribute to its variability. These findings are consistent with previous studies showing decreased circulating aspartate levels in individuals with higher PFAS exposure [82], as well as studies identifying overrepresentation of the aspartate metabolism pathway in populations with elevated PFAS concentration in both sexes [90]. L-aspartic acid participates in key hepatic processes, including amino acid transamination reactions and the urea cycle, which are central to systemic nitrogen balance. In this context, the observed inverse association between PFAS exposure and circulating L-aspartic acid may reflect PFAS-associated alterations in hepatic amino acid metabolism or nitrogen balance. However, this interpretation remains speculative. This framing is consistent with prior human studies reporting PFAS-associated alterations in amino acid metabolic pathways, including arginine, proline, aspartate, and asparagine metabolism [95], as well as perturbations in amino acid pathways linked to the urea cycle [96].

PG 18:0_18:2 also showed moderate discriminatory performance (AUC = 0.653), demonstrating a moderate effect size (Cohen’s d = 0.50) and a weak positive correlation (r = 0.34) with PFAS levels. These results suggest that this glycerophospholipid is involved in PFAS-induced metabolic disruptions. PG are important in the biosynthesis of cardiolipins to maintain mitochondrial functions [97]. Although PG (18:0_18:2) is distinct from PG (16:0/18:1), previously reported as strongly related to PFHpS exposure metabolites [98], both lipids illustrate that PFAS can influence glycerophospholipid metabolism. Supporting this, studies in zebrafish have reported elevated PG levels following PFAS exposure [99] suggesting the occurrence of oxidative damage.

On the other hand, HexCer (d18:1/14:0) demonstrated significant discriminatory performance between the high- and low-exposure groups (AUC = 0.623). However, its very weak and non-significant correlation with PFAS levels (r = 0.151) suggests that the changes in this lipid may not be directly caused solely by PFAS exposure. Instead, this HexCer could reflect broader alterations in lipid metabolism triggered by other physiological factors. In previous studies, in individuals with NAFLD, PFAS exposure was associated with liver metabolites in a gender-specific manner. In females, serum levels of PFOA and PFOS showed positive correlations with ceramides, including Cer(d18:0/16:0) and HexCer(d18:1/18:0) [100]. Comparable trends were observed in mouse models, where PFOA exposure led to elevated levels of Cer and HexCer [100]. PE 16:0_18:3 and TG 16:0_20:5_22:6 showed weaker correlations with PFAS. Despite having AUC values just above 0.6 (0.617 and 0.616, respectively), PE 16:0_18:3 showed a weak positive correlation with PFAS (r = 0.23), suggesting that it might play a role in lipid metabolism responses to PFAS exposure. In a Diabetes Prevention Program trial, the phosphatidylethanolamines subgroup, and more specifically five phosphatidylethanolamines (PE 34:2, PE 36:2, PE 36:3, PE 36:4, and PE 38:4), was significantly associated with total PFOS, l-PFOS, total PFOA, l-PFOA, and br-PFOA [101].

In contrast, TG 16:0_20:5_22:6 showed a very weak, non-significant correlation with PFAS (r = −0.131), indicating a lack of strong univariate association. Nevertheless, its inclusion in the metabolite panel is justified by its multivariate contribution, which enhances the overall discrimination between exposure groups. Previous studies have reported both negative and positive associations between TGs and PFAS, with these inconsistencies likely reflecting differences in study populations [102].

Sensitivity analyses indicated that age, sex, BMI, and blood collection time had minimal influence on the observed associations, supporting the robustness of the panel findings.

The five panel metabolites demonstrated moderate-to-strong effect sizes (Cohen’s d from |−0.84| to |0.4|) and sufficient statistical power to reliably capture exposure-associated alterations within the cohort. However, some of the 38 altered metabolites exhibited smaller effect sizes. Power analyses indicate that larger cohorts (*n* ≥ 150 per group) would allow reliable detection of smaller effect sizes (d ~0.2–0.3).

Overall, this study benefits from a pronounced exposure contrast, with serum PFAS concentrations in the high-exposure group substantially exceeding the background levels reported for the Belgian general population, thereby enhancing the ability to detect exposure-associated metabolic perturbations and increases confidence in the observed associations. The consistency of the identified lipid-centered pathways—particularly glycerophospholipid and sphingolipid metabolism—with prior human, animal, and in vitro studies supports the biological plausibility of the findings and suggests that the observed alterations reflect systemic metabolic responses to PFAS exposure. These pathways are influenced by multiple physiological drivers, indicating that the observed changes are likely PFAS exposure-associated rather than being purely exposure-specific.

A small panel of five metabolites captured interrelated metabolic patterns associated with PFAS exposure, showing moderate discriminatory performance and providing exploratory insights into coordinated metabolic responses rather than serving as a validated biomarker classifier. While some metabolites demonstrated moderate-to-strong discriminatory performances and effect sizes, others exhibited weaker associations, consistent with the idea that certain metabolic changes may reflect broader systemic metabolic responses alongside more specific lipid perturbations. These findings suggest that including such metabolites or lipidomic markers in future HBM studies could aid the identification of early biological responses to PFAS exposure, although further research is needed to confirm their suitability as effect indicators.

Several considerations should be noted. Although this study is cross-sectional and observational, PFAS are highly persistent in the human body, and a single serum measurement in adults likely reflects long-term exposure that precedes metabolic alterations. Therefore, the observed associations are consistent with the expected temporal sequence, even though causal relationships cannot be formally confirmed within this study design. Serum metabolomics provides a systemic snapshot of circulating metabolites, offering a comprehensive view of metabolic responses, though it may not fully capture tissue-specific effects. Some individual-level metadata was self-reported or unavailable, including key lifestyle and clinical covariates such as medication use, smoking, alcohol consumption, diet, and metabolic disorders, potentially contributing to variability in metabolomic profiles and limiting causal inference. For example, statin use can modulate lipid metabolism [103] and alcohol intake can influence circulating amino acids [104], which may influence the observed associations. Samples were collected without standardized fasting, introducing potential circadian and postprandial variability, particularly in circulating lipids and amino acids [105]. However, differences in collection time between exposure groups were small, and the study design reflects typical physiological conditions. Despite the potential for variability introduced by non-fasting status, the observed PFAS-associated metabolic differences appear robust. This is further supported by sensitivity analyses adjusted for age, sex, BMI, and blood collection time, which showed the minimal influence of these covariates on the observed associations. Accordingly, these results should still be interpreted as exploratory contrasts between low- and high-PFAS-exposure groups, with further studies needed to confirm the findings under more controlled conditions.

Despite these limitations, the study provides insights with potential clinical and translational relevance. Lipid-centered metabolic alterations may represent early systemic responses to PFAS exposure and could inform biomonitoring strategies, mechanistic investigations, and risk assessment frameworks. Observed perturbations in amino acid metabolism, lipid signaling, and autophagy-related pathways may help to guide future mechanistic and intervention studies exploring the biological impact of PFAS.

Future research should incorporate longitudinal designs, more comprehensive clinical metadata, and functional approaches that move beyond static metabolite measurements, including metabolic flux analyses and enzymatic activity markers, to clarify the functional significance of observed perturbations. Integrating targeted metabolomics and complementary multi-omics approaches, such as transcriptomics and proteomics, will be essential to disentangle PFAS-specific effects from potential confounding influences and provide a more mechanistically informed and tissue-relevant interpretation of PFAS-associated metabolic dysregulation.

## 5. Conclusions

This study demonstrates that high PFAS exposure in the adult Flemish population residing near a PFAS production facility is associated with coordinated alterations in serum lipid and amino acid profiles, particularly involving glycerophospholipid and sphingolipid metabolism. A small panel of five metabolites, including L-aspartic acid, PG 18:0_18:2, HexCer (d18:1/14:0), PE 16:0_18:3, and TG 16:0_20:5_22:6, captured a moderate discriminatory performance between high- and low-PFAS-exposure groups, which highlighted PFAS-associated metabolic perturbations and may reflect mechanistic links to downstream biological outcomes, providing insight into pathways potentially contributing to broader health effects. Future studies integrating multi-omics approaches and analyses in larger cohorts will be essential to validate these metabolic responses and further elucidate their biological consequences.

## Figures and Tables

**Table 1:** Main characteristics of the study cohort including sex, age, body mass index (BMI), and total PFAS concentration in serum. Sex distribution is reported as the number and percentage of males and females in each group, and the other characteristics as median (Q_1_–Q_3_) for each group.

Characteristics	Low-PFAS-Exposure Group (*n* = 41)	High-PFAS-Exposure Group (*n* = 41)	*p*-Value
Sex—male, *n* (%)	10 (24%)	17 (41%)	0.16 ^1^
Sex—female, *n* (%)	31 (76%)	24 (59%)	
Age (years)	41.3 (37.1–47.4)	48.3 (34.7–57.0)	0.06 ^2,3^
BMI (kg/m^2^)	26.0 (24.0–31.6)	26.6 (23.1–29.1)	0.47 ^2^
Total PFAS serum concentration (ng/mL) ^4^	7.2 (5.9–8.0)	162.0 (110.4–246.6)	˂0.01 ^2^

^1^ χ^2^ test; ^2^ Mann–Whitney U-test; ^3^ Cliff’s delta test = −0.239, 95% CI [−0.462, 0.013]. ^4^ Total PFAS serum concentration was calculated as the sum of the individual PFAS compound concentrations measured in serum, including perfluorobutanoic acid (PFBA), perfluoropentanoic acid (PFPeA), perfluorohexanoic acid (PFHxA), perfluoroheptanoic acid (PFHpA), perfluorooctanoic acid (PFOA), perfluorononanoic acid (PFNA), perfluorodecanoic acid (PFDA), perfluoroundecanoic acid (PFUnA), perfluorododecanoic acid (PFDoA), perfluorobutanesulfonate (PFBS), perfluorohexanesulfonate (PFHxS), perfluoroheptanesulfonate (PFHpS) and perfluorooctanesulfonate (PFOS).

**Table 2:** Evaluation of multivariate model performance. The PLS-DA model was evaluated using the area under the curve (AUC), R^2^, Q^2^ and permutation test (prediction accuracy during training), which was calculated after 1000 random permutations. The random forest (RF) classification model was evaluated by the AUC.

		Polar ESI (+)	Polar ESI (−)	Apolar ESI (+)	Apolar ESI (−)
PLS-DA	AUC	0.70	0.72	0.72	0.78
	R^2^	0.37	0.39	0.30	0.66
	Q^2^	0.23	0.21	0.14	0.36
	PERM (*p*-value)	0.153 ^1^	0.069 ^1^	0.030	0.014
RF	AUC	0.7	0.7	0.7	0.7

^1^ The PLS-DA models for the polar platforms showed non-significant permutation test (*p* > 0.05) and were therefore not used for feature selection.

**Table 3:** Topological impact scores for lipid pathways altered between high- and low-PFAS-exposure groups. Pathway analysis was performed using pathway analysis module of MetaboAnalyst [67], *p*-value, false discovery rate (FDR) [68], and impact on the metabolic pathway are reported.

Pathway	No. Pathway Lipids	Converted Lipids (KEGG IDs)	*p*-Value	FDR	Impact	Effect
Glycerophospholipid metabolism	36	C01194, C03819, C05973, C04438, C00157, C00350	6.9 × 10^−9^	5.6 × 10^−7^	0.2411	Altered
Sphingolipid metabolism	32	C12126, C00195, C00550	5.7 × 10^−4^	0.0228	0.3133	Altered

**Table 4:** Metabolites significantly associated with total PFAS serum concentration.

Metabolite	Correlation (r) ^1^	*p*-Value	Association
PE 16:0_16:1	0.61	0.0236	Positive
PG 18:0_18:2	0.34	0.0031	Positive
PG 18:0_18:1	0.25	0.0308	Positive
PE 16:0_18:3	0.23	0.0476	Positive
SM 18:1/24:2	−0.25	0.0292	Negative
L-Aspartic acid	−0.27	0.0221	Negative
SM 18:1/18:0	−0.31	0.0075	Negative
SM 18:1/22:1	−0.33	0.0036	Negative

^1^ Spearman rank correlation. Positive associations indicate higher metabolite levels with higher total PFAS serum concentration; negative associations indicate lower metabolite levels with higher total PFAS serum concentration. Only significant correlations (*p* < 0.05) are shown.

## Data Availability

The original contributions presented in this study are included in the article/Supplementary Materials. Further inquiries can be directed to the corresponding authors.

## Supplementary Materials

The following supporting information can be downloaded at: https://www.mdpi.com/article/10.3390/metabo16020135/s1, Table S1: Data acquisition parameters; Table S2: MSConvert and MZmine parameters used during data processing; Table S3: MS-FLO parameters; Table S4: Median relative standard deviation (mRSD) (%) of the intensity of LC-MS features for each sample fraction; Table S5: Annotated polar metabolites of the selected features that showed changes between PFAS-exposure groups; Table S6: Annotated lipids of the selected features that showed changes between PFAS-exposure groups; Table S7: Altered lipid pathways and contributing lipids identified through pathway enrichment analysis; Table S8: Exploratory discriminatory performance of individual compounds for high- versus low-PFAS-exposure groups; Table S9: Key metrics for the panel metabolites, including individual AUC, fold change, Cohen’s d, and correlation with total PFAS concentration; Figure S1. Graphical representation of the serum preparation. Figure S2: Principal component analysis plots of serum from PFAS-exposure groups, including quality control (QC) samples, analyzed in different ionization modes (ESI (+) and ESI (−)). Figure S3: Boxplots showing the five metabolites that constitute the panel with a distribution of quantitative responses detected for all individuals; Figure S4. Cohen’s d for the five metabolites that constitute the panel.

## Abbreviations

The following abbreviations are used in this manuscript:

AUC	Area under the curve
BD	Becton, Dickinson and Company
BH	Benjamini–Hochberg
BMI	Body mass index
CAR 3:0	Propionylcarnitine
Cer	Ceramide
CHCl_3_	Chloroform
CL	Confidence level
DDA	Data-dependent acquisition
DG	Diacylglycerol
DTIM	Drift tube-ion mobility
EtNP	Ethanolamine phosphate
ESI	Electrospray ionization
FC	Fold change
FDR	False discovery rate
GPI	Glycosylphosphatidylinositol
H_2_O	Water
HCOOH	Formic acid
HexCer	Hexosylceramide
IPA	Isopropanol
IS	Internal standard
LC-HRMS	Liquid chromatography–high-resolution mass spectrometry
LLE	Liquid–liquid extraction
LOD	Limit of detection
LOQ	Limit of quantification
LPE	Lysophosphatidylethanolamine
LPI	Lysophosphatidylinositol
MeCN	Acetonitrile
MeOH	Methanol
MPA	Mobile phase A
MPB	Mobile phase B
NAPE	N-acyl-phosphatidylethanolamine
PC	Phosphatidylcholine
PCA	Principal component analysis
PE	Phosphatidylethanolamine
PG	Phosphatidylglycerol
PI3P	Phosphatidylinositol-3-phosphate
PI	Phosphatidylinositol
PFAS	Per- and polyfluoroalkyl substances
PFBA	Perfluorobutanoic acid
PFBS	Perfluorobutanesulfonate
PFDA	Perfluorodecanoic acid
PFDoA	Perfluorododecanoic acid
PFHpA	Perfluoroheptanoic acid
PFHpS	Perfluoroheptanesulfonate
PFHxA	Perfluorohexanoic acid
PFHxS	Perfluorohexanesulfonate
PFOA	Perfluorooctanoic acid
PFOS	Perfluorooctanesulfonate
PFNA	Perfluorononanoic acid
PFPeA	Perfluoropentanoic acid
PFUnA	Perfluoroundecanoic acid
PLS-DA	Partial least squares-discriminant analysis
PQN	Probabilistic quotient normalization
QC	Quality control
RF	Random forest
ROC	Receiver operating characteristic
RSD	Relative standard deviation
mRSD	Median RSD
RT	Retention time
SM	Sphingomyelin
S1P	Sphingosine-1-phosphate
TG	Triacylglycerol
VIFs	Variance inflation factors
