# Supplementary material for: Untargeted Metabolomics Profiling of a PFAS-Exposed Flemish Population
Source: Metabolites. 2026 Feb 15;16(2):135. doi: 10.3390/metabo16020135 (PMC12943362; doi:10.3390/metabo16020135)
Supplement: Supplementary file 1 [file metabolites-16-00135-s001.zip › metabolites-4128639-supplementary.pdf]

metabolites

MDPI

Article

Untargeted metabolomics profiling of a PFAS-exposed Flemish population

María del Mar Delgado-Povedano [1,*], Haesong Sher [1], Leen Jacobs [1], Maria van de Lavoir [1], Rani Robeyns [1], Ann Colles [2], Eva Govarts [2], Elly Den Hond [3], Giulia Poma [1], Alexander L.N. van Nuijs [1] and Adrian Covaci [1,*]

[1] Toxicological Centre, University of Antwerp, 2610 Antwerp, Belgium
[2] VITO Health, Flemish Institute for Technological Research (VITO), Boeretang 200, 2400 Mol, Belgium
[3] Provincial Institute of Hygiene, Provincial Research Centre for Environment and Health, 2023 Antwerp, Belgium

* Correspondence: M.D.P., mariadelmar.delgadopovedano@uantwerpen.be; A.C. adrian.covaci@uantwerpen.be

Supplementary Materials

TABLE OF CONTENTS

Table S1. Data acquisition parameters.
Table S2. MSConvert and MZmine parameters used during data processing.
Table S3. MS-FLO parameters.
Table S4. Median relative standard deviation (mRSD) (%) of the intensity of LC-MS features for each sample fraction.
Table S5. Annotated polar metabolites of the selected features that showed changes between PFAS exposure groups.
Table S6: Annotated lipids of the selected features that showed changes between PFAS exposure groups.
Table S7. Altered lipid pathways and contributing lipids identified through pathway enrichment analysis.
Table S8. Exploratory discriminatory performance of individual compounds for high versus low PFAS exposure groups.
Table S9. Key metrics for the panel metabolites, including individual AUC, fold change, Cohen's d, and correlation with total PFAS concentration.

Figure S1. Graphical representation of the serum preparation.
Figure S2. Principal component analysis plots of serum from PFAS exposure groups, including quality control (QC) samples, analyzed in different ionization modes (ESI (+) and ESI (−)).
Figure S3. Boxplots showing the five metabolites that constitute the panel with a distribution of quantitative responses detected for all individuals.
Figure S4. Cohen's de for the five metabolites that constitute the panel.

 https://doi.org/10.3390/metabo16020135

Table S1. Data acquisition parameters. For polar methods (ESI (+) and ESI (-)), additional MS2 runs were acquired using one collision energy at a time (10, 20 or 40 eV) with a maximum of 12 precursors per scan cycle*. BEH: Ethylene bridged hybrid. ESI: Electrospray ionization. LC: Liquid chromatography. UPLC: Ultra performance liquid chromatography. MeOH: Methanol. MeCN: Acetonitrile. IPA: Isopropanol. QToF: Quadrupole-time-of-flight.

Sample fraction		Polar ESI (+)	Polar ESI (-)	Apolar ESI (+)	Apolar ESI (-)
LC	System	Agilent 1290 Infinity	Agilent 1290 Infinity	Agilent 1290 Infinity II	Agilent 1290 Infinity II
	Column (dimensions)	iHILIC-Fusion (100 x 2.1 mm, 1.8 µm)	iHILIC-Fusion(P) (100 x 2.1 mm, 5 µm)	ACQUITY UPLC BEH C18 (50 × 2.1 mm, 1.7 µm)	ACQUITY UPLC BEH C18 (50 × 2.1 mm, 1.7 µm)
	Mobile Phase A (MPA)	10 mM $HCOONH_4$ + 0.1% (v/v) HCOOH in H_2O/MeOH (9/1, v/v)	2 mM CH_3COONH_4 + 2 mM $(NH_4)_2CO_3$ in H_2O	5 mM CH_3COONH_4 + 0.1% (v/v) CH_3COOH in H_2O/MeCN (7/3, v/v)	5 mM CH_3COONH_4 in H_2O/MeCN (7/3, v/v)
	Mobile Phase B (MPB)	MeCN	MeCN/MeOH (9/1, v/v)	5 mM CH_3COONH_4 + 0.1% (v/v) CH_3COOH in H_2O/MeCN/IPA (2/10/88, v/v/v)	5 mM CH_3COONH_4 in H_2O/MeCN/IPA (2/10/88, v/v/v)
	Gradient	Time (min) – % MPB	Time (min) – % MPB	Time (min) – % MPB	Time (min) – % MPB
		0–95	0–95	0–15	0–15
		4–95	1–95	0.7–15	0.7–15
		12.5–60	10–20	1.0–30	1.0–30
		20–60	14–20	1.7–60	1.7–60
		21–95	15–95	2.7–60	2.7–60
		26–95	20–95	6.7–100	6.7–100
				12.0–100	12.0–100
				14.0–15	14.0–15
				16.0–15	16.0–15
	Temperature (°C)	60	25, bypassing heat exchanger	60	60
	Flow rate (mL/min)	0.25	0.2	0.2	0.2

	Injection volume (µL)	3	3	2	2
MS	System	Agilent 6530 QToF	Agilent 6530 QToF	Agilent 6560 (DTIM)-QToF	Agilent 6560 (DTIM)-QToF
	Acquisition	2 GHz extended dynamic mode	2 GHz extended dynamic mode	2 GHz extended dynamic mode	2 GHz extended dynamic mode
	Data-dependent acquisition	AutoMS/MS with active exclusion	AutoMS/MS with active exclusion	AutoMS/MS with active exclusion<br>Iterative exclusion MS/MS	AutoMS/MS with active exclusion<br>Iterative exclusion MS/MS
	MS1 range (Da)	60–1200	60–1200	100–1700	100–1700
	MS2 range (Da)	40–1000	40–1000	70–1700	70–1700
	Scan rate MS1 (spectra/s)	2	2	3	3
	Scan rate MS2 (spectra/s)	6	6	6	6
	Nr. of precursors/cycles	4*	4*	6	6
	Quadrupole isolation window	Narrow (1.3 amu)	Narrow (1.3 amu)	Narrow (1.3 amu)	Narrow (1.3 amu)
	CE (eV)	10, 20, 40*	10, 20, 40*	10, 20, 40	10, 20, 40
ESI					
	Nozzle voltage (V)	0	0	500	500
	Capillary voltage (V)	2000	2000	3500	3750
	Fragmentor voltage (V)	150	100	200	200
	Drying gas	Nitrogen	Nitrogen	Nitrogen	Nitrogen
	Sheath gas	Nitrogen	Nitrogen	Nitrogen	Nitrogen
	Drying gas temperature (°C)	250	250	325	350

Sheath gas temperature (°C)	350	350	325	350
Drying gas flow (L/min)	8	10	8	8
Sheath gas flow (L/min)	11	10	8	8
Nebulizer gas pressure (psig)	45	45	30	30

Table S2. MSConvert and MZmine parameters used during data processing.

The raw LC-HRMS data files, stored in the Agilent .d format, were converted to open-source .mzML format using MSConvert (ProteoWizard, v. 3.0.19317). Conversion was performed with 64-bit binary encoding precision, zlib compression, and index writing enabled, with TPP compatibility selected. Vendor peak picking was applied to profile-mode data. For MS-only files, peak picking was performed at MS level 1, while for MS/MS files, peak picking was applied at MS levels 1–2. Data were converted separately for MS and MS/MS scans. The original ion polarity (positive/negative) was preserved during conversion. All other parameters were kept at default settings.

Sample fraction		Polar ESI (+)	Polar ESI (-)	Apolar ESI (+)	Apolar ESI (-)
MZmine	Crop filter	0.5-22 min<br>60-1200 m/z	0.5-19.5 min<br>60-1200 m/z	0.5-14 min<br>60-1700 m/z	0.5-14 min<br>60-1700 m/z
	Mass detection	MS1, centroid, 400 noise level	MS1, centroid, 500 noise level	MS1, centroid, 400 noise level	MS1, centroid, 400 noise level
	Mass detection	MS2, centroid, 10 noise level	MS2, centroid, 10 noise level	MS2, centroid, 10 noise level	MS2, centroid, 10 noise level
	Chromatogram builder	MS1, 4 minimum consecutive scans, 1200 minimum absolute height, 15 ppm or 0.005 m/z tolerance (scan-to-scan)	MS1, 4 minimum consecutive scans, 1500 minimum absolute height, 15 ppm or 0.005 m/z tolerance (scan-to-scan)	MS1, 4 minimum consecutive scans, 1200 minimum absolute height, 15 ppm or 0.005 m/z tolerance (scan-to-scan)	MS1, 4 minimum consecutive scans, 1200 minimum absolute height, 15 ppm or 0.005 m/z tolerance (scan-to-scan)
	Local minimum feature resolver	15 ppm or 0.005 m/z MS1 to MS2 precursor tolerance.<br>Retention time filter: use feature edges, 3% minimum relative feature height, retention time, 80% chromatographic threshold, 1200 minimum absolute height, 1.7 min ratio of peak top/edge, 4 minimum scans	15 ppm or 0.005 m/z MS1 to MS2 precursor tolerance.<br>Retention time filter: use feature edges, 3% minimum relative feature height, retention time, 80% chromatographic threshold, 1500 minimum absolute height, 1.7 min ratio of peak top/edge, 4 minimum scans	15 ppm or 0.005 m/z MS1 to MS2 precursor tolerance.<br>Retention time filter: use feature edges, 3% minimum relative feature height, retention time, 80% chromatographic threshold, 1200 minimum absolute height, 1.7 min ratio of peak top/edge, 4 minimum scans	15 ppm or 0.005 m/z MS1 to MS2 precursor tolerance.<br>Retention time filter: use feature edges, 3% minimum relative feature height, retention time, 80% chromatographic threshold, 1200 minimum absolute height, 1.7 min ratio of peak top/edge, 4 minimum scans
	13C isotope filter (formerly: isotope grouper)	3.5 ppm or 0.001 m/z tolerance (intra-sample), 0.02 min ΔRT, monotonic shape, 2 maximum charge, most intense representative isotope	3.5 ppm or 0.001 m/z tolerance (intra-sample), 0.02 min ΔRT, monotonic shape, 2 maximum charge, most intense representative isotope	3.5 ppm or 0.001 m/z tolerance (intra-sample), 0.02 min ΔRT, monotonic shape, 2 maximum charge, most intense representative isotope	3.5 ppm or 0.001 m/z tolerance (intra-sample), 0.02 min ΔRT, monotonic shape, 2 maximum charge, most intense representative isotope

	Isotopic peaks finder	H,C,N,O,S,P, 5 ppm or 0.001 m/z tolerance (feature to scan), 2 maximum charge of isotope m/z	H,C,N,O,S,P, 5 ppm or 0.001 m/z tolerance (feature to scan), 2 maximum charge of isotope m/z	H,C,N,O,S,P, 5 ppm or 0.001 m/z tolerance (feature to scan), 2 maximum charge of isotope m/z	H,C,N,O,S,P, 5 ppm or 0.001 m/z tolerance (feature to scan), 2 maximum charge of isotope m/z
	Join aligner	15 ppm or 0.005 m/z tolerance (sample-to-sample), 3 weight for m/z, 0.5 min ΔRT, 1 weight for RT, require same charge state	15 ppm or 0.005 m/z tolerance (sample-to-sample), 3 weight for m/z, 0.2 min ΔRT, 1 weight for RT, require same charge state	15 ppm or 0.005 m/z tolerance (sample-to-sample), 3 weight for m/z, 0.07 min ΔRT, 1 weight for RT, require same charge state	15 ppm or 0.005 m/z tolerance (sample-to-sample), 3 weight for m/z, 0.07 min ΔRT, 1 weight for RT, require same charge state
	Group MS2 scans with features	30 ppm or 0.05 m/z MS1 to MS2 percursor tolerance (m/z). Retention time filter: Use feature edges. 25% minimum relative feature height	30 ppm or 0.05 m/z MS1 to MS2 percursor tolerance (m/z). Retention time filter: Use feature edges. 25% minimum relative feature height	30 ppm or 0.05 m/z MS1 to MS2 percursor tolerance (m/z). Retention time filter: Use feature edges. 25% minimum relative feature height	30 ppm or 0.05 m/z MS1 to MS2 percursor tolerance (m/z). Retention time filter: Use feature edges. 25% minimum relative feature height
	Feature finder (multithreaded)	20% intensity tolerance, 15 ppm or 0.005 m/z tolerance (sample-to-sample), 0.15 min ΔRT, 4 minimum scans	20% intensity tolerance, 15 ppm or 0.005 m/z tolerance (sample-to-sample), 0.15 min ΔRT, 4 minimum scans	20% intensity tolerance, 15 ppm or 0.005 m/z tolerance (sample-to-sample), 0.07 min ΔRT, 4 minimum scans	20% intensity tolerance, 15 ppm or 0.005 m/z tolerance (sample-to-sample), 0.07 min ΔRT, 4 minimum scans
	Duplicate peak filter	4 ppm or 0.001 m/z tolerance, 0.1 minutes RT tolerance	4 ppm or 0.001 m/z tolerance, 0.1 minutes RT tolerance	4 ppm or 0.001 m/z tolerance, 0.07 minutes RT tolerance	4 ppm or 0.001 m/z tolerance, 0.07 minutes RT tolerance
	Correlation grouping (metaCorrelate)	0.1 minutes ΔRT, feature shape correlation (5 min data points, 2 min data points edge, Pearson, 85% min feature shape correlation)	0.1 minutes ΔRT, feature shape correlation (5 min data points, 2 min data points edge, Pearson, 85% min feature shape correlation)	0.04 minutes ΔRT, feature shape correlation (5 min data points, 2 min data points edge, Pearson, 85% min feature shape correlation)	0.04 minutes ΔRT, feature shape correlation (5 min data points, 2 min data points edge, Pearson, 85% min feature shape correlation)
	Ion identity networking	3.5 ppm or 0.01 m/z tolerance (intra-sample), one feature, 1200 min height, $[M+H]^+$, $[M+NH_4]^+$, $[M+Na]^+$, $[M-H_2O+H]^+$	3.5 ppm or 0.01 m/z tolerance (intra-sample), one feature, 1500 min height, $[M-H]^-$, $[M-H_2O-H]^-$, $[M+HCOO]^-$ $[M+CH_3COO]^-$	3.5 ppm or 0.01 m/z tolerance (intra-sample), one feature, 1200 min height, $[M+H]^+$, $[M+NH_4]^+$, $[M+Na]^+$, $[M-H_2O+H]^+$	3.5 ppm or 0.01 m/z tolerance (intra-sample), one feature, 1200 min height, $[M-H]^-$, $[M-H_2O-H]^-$, $[M+HCOO]^-$ $[M+CH_3COO]^-$

	Spectral library search	Merged, 25 ppm or 0.01 merging m/z tolerance, MS2, 20 ppm precursor tolerance, 4 minimum matched signals, weighted cosine similarity, MassBank, 0.8 minimum cos similarity	Merged, 25 ppm or 0.01 merging m/z tolerance, MS2, 20 ppm precursor tolerance, 4 minimum matched signals, weighted cosine similarity, MassBank, 0.8 minimum cos similarity	Merged, 25 ppm or 0.01 merging m/z tolerance, MS2, 20 ppm precursor tolerance, 4 minimum matched signals, weighted cosine similarity, MassBank, 0.8 minimum cos similarity	Merged, 25 ppm or 0.01 merging m/z tolerance, MS2, 20 ppm precursor tolerance, 4 minimum matched signals, weighted cosine similarity, MassBank, 0.8 minimum cos similarity

Table S3. MS-FLO parameters. The peak lists from MZmine were further processed with MS-FLO for additional deisotoping and removal of duplicates. The following parameters were used for peak detection and alignment.

Sample fraction			Polar ESI (+)	Polar ESI (-)	Apolar ESI (+)	Apolar ESI (-)
MS-FLO	Duplicate peak removal	m/z tolerance (Da)	0.01	0.01	0.01	0.01
		RT tolerance (min)	0.1	0.1	0.1	0.1
		Peak height tolerance	500	500	500	500
		Minimum peak match ratio	0.8	0.8	0.8	0.8
	Isotope detection	m/z tolerance (Da)	0.01	0.01	0.01	0.01
		RT tolerance (min)	0.02	0.02	0.02	0.02
		R^2 for isotope match	0.8	0.8	0.8	0.8

Table S4. Median relative standard deviation (mRSD, %) of the intensity of LC-MS features for each sample fraction. mRSD values were calculated after deisotoping and blank subtraction with no gap filling.

	Polar ESI (+) (%)	Polar ESI (–) (%)	Apolar ESI (+) (%)	Apolar ESI (–) (%)
Low exposure	31.0	43.5	32.5	28.8
High exposure	32.3	46.0	31.3	29.4
QC	14.4	14.3	11.1	11.0

Table S5. Annotated polar metabolites of the selected features that showed changes between PFAS exposure groups. Metabolites are classified by subclass and superclass according to RefMet and reported using RefMet shorthand notation. All structures are reported according to the annotation confidence level (CL) system of Schymanski et al. (2014). Level 2 implies a probable structure and is divided into 2a by matching library or literature data, and 2b by diagnostic evidence. Level 1 implies a confirmed structure by matching a reference standard. RT: retention time.

Name (trivial)	Name (Refmet)	HMDB ID	Subclass (refmet)	Superclass (refmet)	Formula	Ion species	m/z	RT (min)	Mass error (ppm)	CL	Library/database match	MS2 fragments matched
L-Aspartic acid	Aspartic acid	HMDB0000191	Amino acids	Organic acids	$C_4H_7NO_4$	[M+H]-	132.0302	5.27	0.2	1	MassBank, GNPS, NIST, Fiehn HILIC, PUBLIC_EXP_NEG_VS17, In-house library	71.0129, 88.0371, 114.0149, 115.0014
CAR (3:0)	CAR 3:0	HMDB0062514	Acyl carnitines	Fatty acyls	$C_{10}H_{19}NO_4$	[M+H]+	218.139	12.36	1.4	2a	Fiehn HILIC, MoNA, NIST, PUBLIC_EXP_POS_VS17	60.0820, 85.0296, 144.1046, 159.0667

Table S6. Annotated lipids of the selected features that showed changes between PFAS exposure groups. Lipids are reported at both the sum-composition (bulk) and molecular species levels using the Liebisch (LIPID MAPS) shorthand lipid notation and classified by subclass according to the LIPID MAPS classification system. RT: retention time.

Bulk level	Molecular species level	Subclass	Formula	Adduct	m/z	RT (min)	Mass error (ppm)
Cer 39:1;2O	Cer (d16:1/23:0)	Ceramides	$C_{39}H_{77}NO_3$	$[M+CH_3COO]^-$	666.6057	7.01	2.3
Cer 40:1;2O	Cer (d18:1/22:0)	Ceramides	$C_{40}H_{79}NO_3$	$[M-H]^-$	620.5990	7.11	0.5
Cer 42:2;3O	Cer 18:1;3O/24:1	Ceramides	$C_{42}H_{81}NO_4$	$[M-H]^-$	662.6090	7.02	-0.5
Cer 43:2;2O	Cer (d19:1/24:1)	Ceramides	$C_{43}H_{83}NO_3$	$[M+CH_3COO]^-$	720.6505	7.17	-0.8

HexCer 32:1;2O	HexCer (18:1/14:0)	Hexosylceramides	$C_{38}H_{73}NO_8$	$[M-H]^-$	670.5272	5.84	1.3
HexCer 42:1;2O	HexCer (d18:1/24:0)	Hexosylceramides	$C_{48}H_{93}NO_8$	$[M+CH_3COO]^-$	870.7032	7.11	-0.9
HexCer 42:2;2O	HexCer (d18:1/24:1)	Hexosylceramides	$C_{48}H_{91}NO_8$	$[M+CH_3COO]^-$	868.6882	6.94	-0.1
LPE 16:0	LPE 16:0	Glycerophospho-ethanolamines	$C_{21}H_{44}NO_7P$	$[M-H]^-$	452.2789	3.61	1.3
LPI 18:2	LPI 18:2	Phosphatidylinosi-tols	$C_{27}H_{49}O_{12}P$	$[M-H]^-$	595.2884	2.99	-0.8
PC 36:1	PC 18:0_18:1	Glycerophospho-cholines	$C_{44}H_{86}NO_8P$	$[M+CH_3COO]^-$	846.6230	6.66	0.0
PC 36:3	PC 16:0_20:3	Glycerophospho-cholines	$C_{44}H_{82}NO_8P$	$[M+CH_3COO]^-$	842.5902	6.28	-1.8
PC 36:5;2O	PC 14:0_22:5;2O	Glycerophospho-cholines	$C_{44}H_{78}NO_{10}P$	$[M+H]^+$	812.5435	4.72	-0.1
PE 32:1	PE 16:0_16:1	Glycerophospho-ethanolamines	$C_{37}H_{72}NO_8P$	$[M-H]^-$	688.4920	6.19	-0.4
PE 34:0	PE 16:0_18:0	Glycerophospho-ethanolamines	$C_{39}H_{78}NO_8P$	$[M-H]^-$	718.5396	6.70	0.6
PE 34:1	PE 16:0_18:1	Glycerophospho-ethanolamines	$C_{39}H_{76}NO_8P$	$[M-H]^-$	716.5224	6.47	-1.7
PE 34:3	PE 16:0_18:3	Glycerophospho-ethanolamines	$C_{39}H_{72}NO_8P$	$[M-H]^-$	712.4930	6.03	1.0
PE 36:3	PE 18:1_18:2	Glycerophospho-ethanolamines	$C_{41}H_{76}NO_8P$	$[M-H]^-$	740.5234	6.28	-0.3
PE 37:6	PE 15:0_22:6	Glycerophospho-ethanolamines	$C_{42}H_{72}NO_8P$	$[M-H]^-$	748.4935	6.00	1.6

PE 38:4	PE 18:0_20:4	Glycerophospho-ethanolamines	$C_{43}H_{78}NO_8P$	[M-H]$^-$	766.5397	6.49	0.7
PE 40:7	PE 18:1_22:6	Glycerophospho-ethanolamines	$C_{45}H_{76}NO_8P$	[M-H]$^-$	788.5240	6.18	0.5
PG 36:1	PG 18:0_18:1	Phosphatidylglycer-ols	$C_{42}H_{81}O_{10}P$	[M-H]$^-$	775.5493	5.73	-0.3
PG 36:2	PG 18:0_18:2	Phosphatidylglycer-ols	$C_{42}H_{79}O_{10}P$	[M-H]$^-$	773.5330	5.56	-1.0
PI 32:0	PI 16:0/16:0	Phosphatidylinosi-tols	$C_{41}H_{79}O_{13}P$	[M-H]$^-$	809.5183	5.38	-0.4
PI 32:1	PI 16:0_16:1	Phosphatidylinosi-tols	$C_{41}H_{77}O_{13}P$	[M-H]$^-$	807.5022	5.13	-0.9
PI 35:1	PI 17:0_18:1	Phosphatidylinosi-tols	$C_{44}H_{83}O_{13}P$	[M-H]$^-$	849.5495	5.57	-0.5
PI 36:2	PI 18:0_18:2	Phosphatidylinosi-tols	$C_{45}H_{83}O_{13}P$	[M-H]$^-$	861.5497	5.51	-0.2
PI 37:4	PI 17:0_20:4	Phosphatidylinosi-tols	$C_{46}H_{81}O_{13}P$	[M-H]$^-$	871.5333	5.35	-1.0
PI 39:4	PI 19:0_20:4	Phosphatidylinosi-tols	$C_{48}H_{85}O_{13}P$	[M-H]$^-$	899.5643	5.62	-1.3
SM 33:1		Sphingomyelins	$C_{38}H_{77}N_2O_6P$	[M+CH$_3$ COO]$^-$	747.5654	5.89	-0.5
SM 36:1	SM 18:1/18:0	Sphingomyelins	$C_{41}H_{83}N_2O_6P$	[M+CH$_3$ COO]$^-$	789.6118	6.37	-1.1
SM 38:1	SM 18:1/20:0	Sphingomyelins	$C_{43}H_{87}N_2O_6P$	[M+CH$_3$ COO]$^-$	817.6437	6.65	-0.4
SM 40:2	SM 18:1/22:1	Sphingomyelins	$C_{45}H_{89}N_2O_6P$	[M+CH$_3$ COO]$^-$	843.6596	6.71	-0.1
SM 42:3	SM 18:1/24:2	Sphingomyelins	$C_{47}H_{91}N_2O_6P$	[M+CH$_3$ COO]$^-$	869.6748	6.71	-0.6
TG 52:3;O	TG 16:0_18:1_18:2;O	Triradylglycerols	$C_{55}H_{100}O_7$	[M+NH$_4$]$^+$	890.7773	7.48	-3.8

TG 58:11	TG 16:0_20:5_22:6	Triradylglycerols	$C_{61}H_{96}O_6$	$[M+NH_4]^+$	942.7579	7.53	3.6
TG 60:12		Triradylglycerols	$C_{63}H_{98}O_6$	$[M+NH_4]^+$	968.7742	7.59	4.1

Table S7. Altered lipid pathways and contributing lipids identified through pathway enrichment analysis. FDR: Fase Discovery Rate.

**Pathway**	**No. Pathway lipids**	**Converted lipids[1] (KEGG IDs)**	**p-value**	**FDR**
Glycerophospholipid metabolism	26	C01194, C03819, C05973, C04438, C00157, C00350	1.64E-05	0.0004
Sphingolipid metabolism	21	C12126, C00195, C00550	0.0142	0.0409
Sphingolipid signaling pathway	9	C00550, C12126, C00195	0.0011	0.0095
Retrograde endocannabinoid signaling	8	C00350, C00157	0.0162	0.0413
Glycosylphosphatidylinositol-anchor biosynthesis	3	C00350, C01194	0.0019	0.0095
Autophagy	4	C00350, C01194	0.0037	0.0121
Necroptosis	4	C00195, C00550	0.0037	0.0121

[1] Lipids list conversion (by LIPEA)

**Type**	**Format**	**List**
Original lipids	Abbreviation	PE 37:6, PI 32:0, PE 32:1, TG 52:3;O, PI 32:1, CAR (3:0), PE 34:3, PI 35:1, PE 40:7, PG 36:2, PG 36:1, HexCer 32:1;2O, Cer 43:2;2O, Cer 39:1;2O, PE 34:1, LPE 16:0, PE 36:3, HexCer 42:1;2O, PE 34:0, SM 33:1, PI 37:4, PI 39:4, PE 38:4, PI 36:2, PC 36:1, Cer 40:1;2O, HexCer 42:2;2O, PC 36:3, Cer 42:2;3O, SM 38:1 , SM 42:3, SM 40:2, LPI 18:2, SM 36:1, TG 60:12, TG 58:11, PC 36:5;2O
Converted lipids	KEGG IDs	C00157, C21480, C00350, C04438, C05973, C13883, C00626, C01194, C03819, C00195, C12126, C00550

Table S8. Exploratory discriminatory performance of individual compounds for high versus low PFAS exposure groups. AUC: area under the curve. FC: Fold change.

Metabolite	AUC[1] (95% CI)	t-test (p-value)	log_2FC
L-Aspartic acid	0.722 (0.593–0.829)	0.0006	-0.39
SM 18:1/22:1	0.673 (0.551–0.795)	0.0156	-0.14
SM 18:1/18:0	0.654 (0.521–0.786)	0.0304	-0.20
PG 18:0_18:2	0.653 (0.522–0.787)	0.0366	0.20
PI 17:0_18:1	0.619 (0.482–0.741)	0.0451	0.27
HexCer (d18:1/14:0)	0.623 (0.500–0.743)	0.0421	0.16
SM 18:1/20:0	0.626 (0.510–0.742)	0.0447	-0.11

[1] AUC = area under the ROC curve. Only metabolites with statistically significant discriminatory performance ($p < 0.05$) are shown.

Table S9. Key metrics for the panel metabolites, including individual AUC, log_2 FC, Cohen's d, and correlation with total PFAS concentration. AUC: area under the curve. FC: Fold change.

Metabolite	AUC (95% CI)	log_2FC	Cohen's d	Correlation (r)
L-Aspartic acid	0.722 (0.593–0.829)	-0.39	-0.84	-0.27
PG 18:0_18:2	0.653 (0.522–0.787)	0.20	0.50	0.34
HexCer (d18:1/14:0)	0.623 (0.500–0.743)	0.39	0.48	0.15[1]
TG 16:0_20:5_22:6	0.617 (0.486–0.793)[1]	-0.51	-0.43[1]	-0.13[1]
PE 16:0_18:3	0.616 (0.482–0.737)[1]	0.33	0.40[1]	0.23

[1] Not statistically significant (p-value > 0.05)

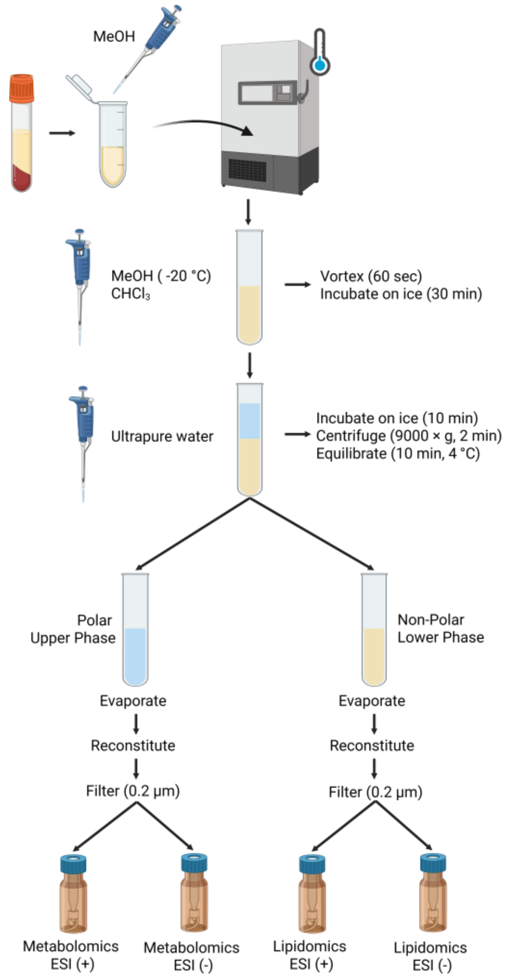

Figure S1. Graphical representation of the serum preparation. Liquid-liquid extraction was performed using MeOH/H_2O/$CHCl_3$ (4:3;8:, v/v/v), generating polar and apolar fractions. Each fraction was divided into two subfractions for analysis in ESI (+) and ESI (−) modes. MeOH: Methanol. $CHCl_3$: chloroform. The figure was created in BioRender. sher, H. (2026) https://BioRender.com/0b5sear.

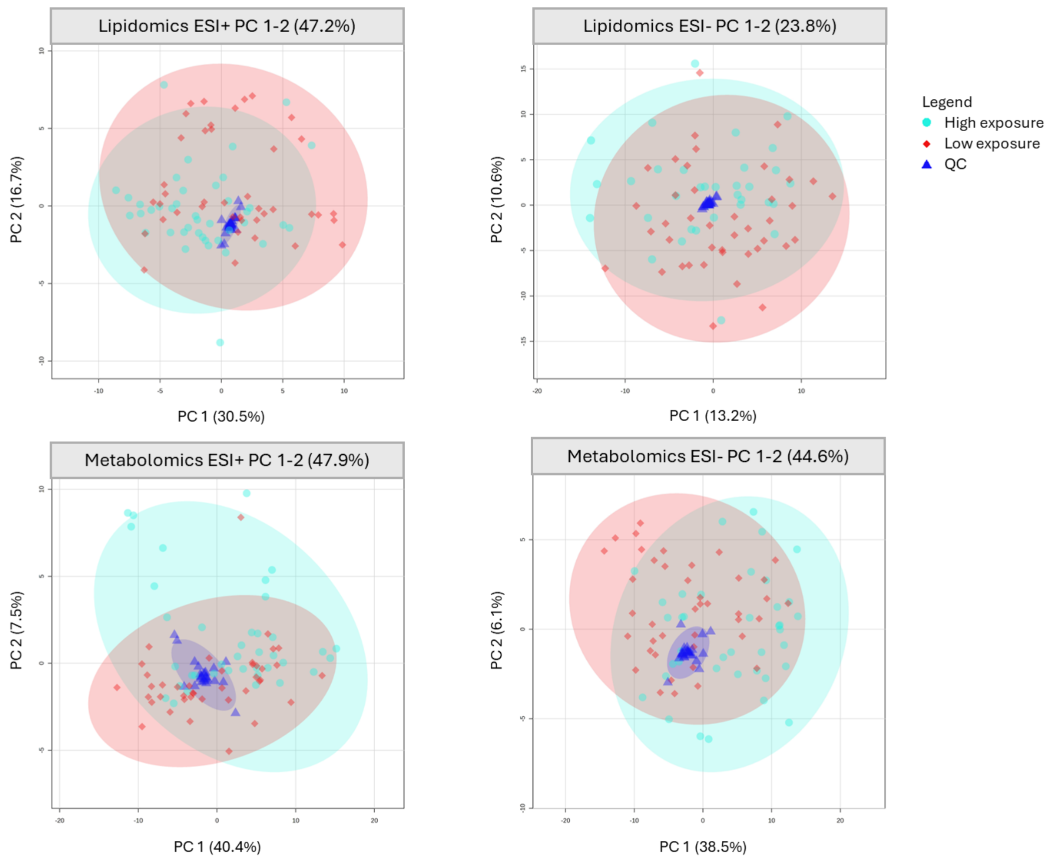

Figure S2. Principal component analysis plots of serum from PFAS exposure groups, including quality control (QC) samples, analyzed in different ionization modes (ESI (+) and ESI (–)). Lipidomics plots refer to the apolar sample fraction, and metabolomics plots refer to the polar sample fraction. There is a partial separation between the high exposure group (green) and the low exposure group (red), indicating some inter-group variability. The clustering of QC samples (dark blue) demonstrates good instrument reproducibility.

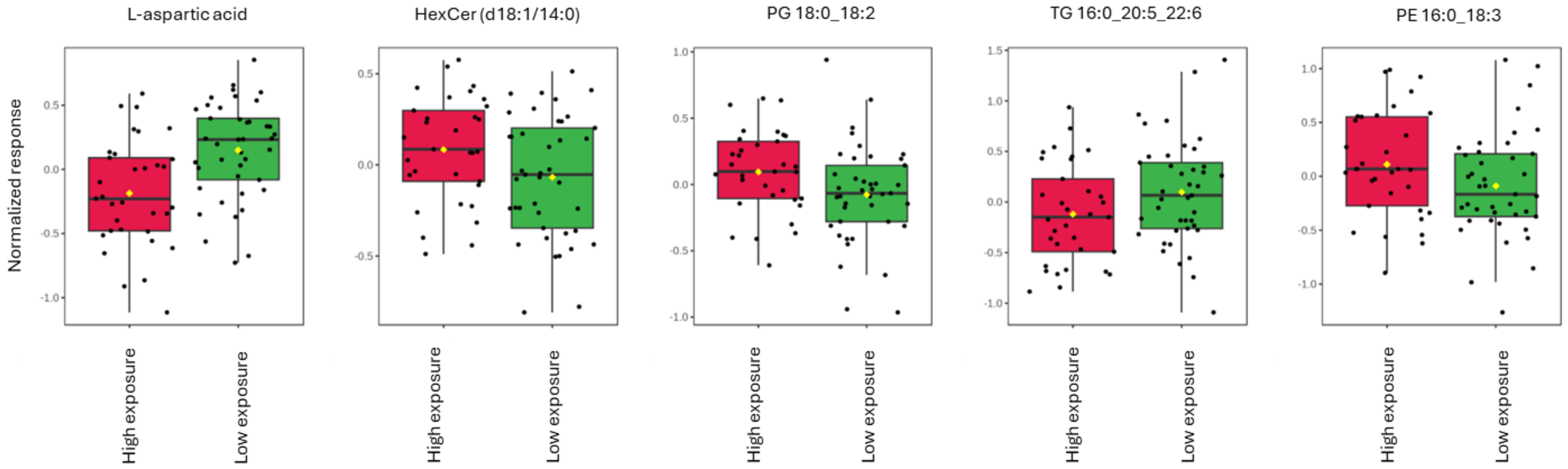

Figure S3. Boxplots showing the five metabolites that constitute the panel with a distribution of normalized responses detected for all individuals.

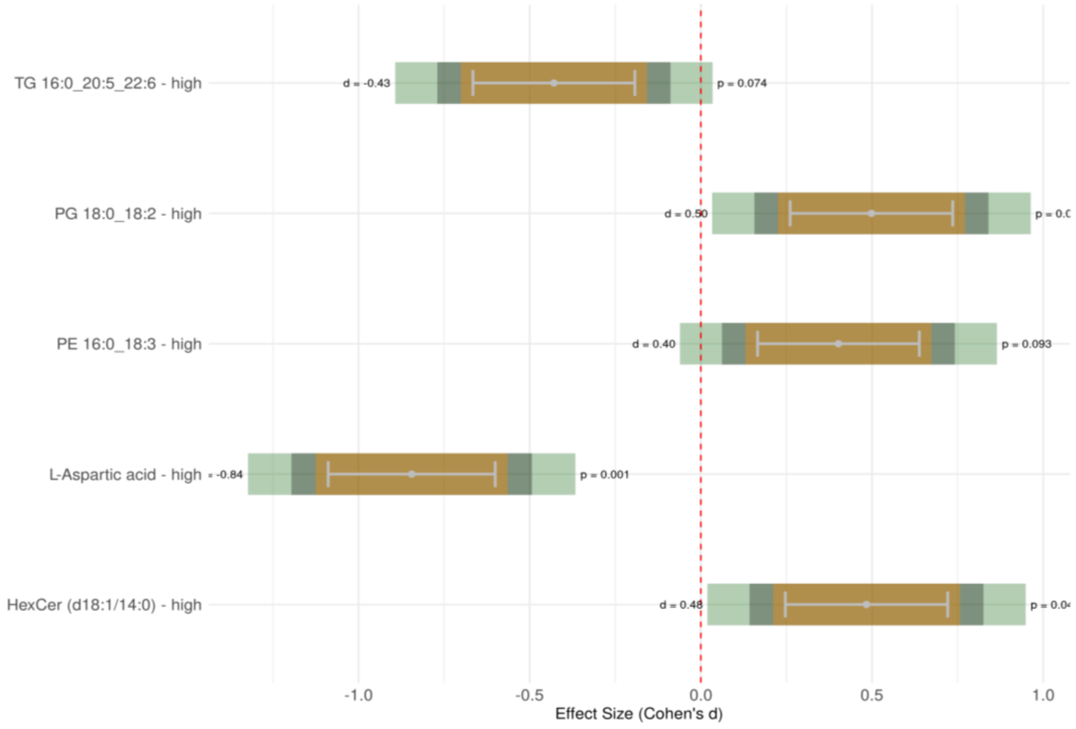

Figure S4. Cohen's d for the five metabolites that constitute the panel.
